# Cellulose Acetate Nanofibers: Incorporating Hydroxyapatite (HA), HA/Berberine or HA/Moghat Composites, as Scaffolds to Enhance In Vitro Osteoporotic Bone Regeneration

**DOI:** 10.3390/polym13234140

**Published:** 2021-11-27

**Authors:** Nadia Z. Shaban, Marwa Y. Kenawy, Nahla A. Taha, Mona M. Abd El-Latif, Doaa A. Ghareeb

**Affiliations:** 1 Biochemistry Department, Faculty of Science, Alexandria University, Alexandria 21511, Egypt; nshaban2001@yahoo.co.uk; 2 Fabrication Technology Researches Department, Advanced Technology and New Materials Research Institute (ATNMRI), City of Scientific Research and Technological Applications (SRTA-City), New Borg El-Arab, Alexandria 21934, Egypt; amona1911@yahoo.com; 3 Modeling and Simulation Research Department, Advanced Technology and New Materials Research Institute (ATNMRI), City of Scientific Research and Technological Applications (SRTA-City), New Borg El-Arab, Alexandria 21934, Egypt; nahlataha_1982@yahoo.com; 4 Bio-Screening and Preclinical Trial Lab, Biochemistry Department, Faculty of Science, Alexandria University, Alexandria 21511, Egypt; 5 Center of Excellence for Drug Preclinical Studies (CE-DPS), Pharmaceutical and Fermentation Industries Development Center (PFIDC), City of Scientific Research & Technological Applications (SRTA-City), New Borg El Arab, Alexandria 21934, Egypt

**Keywords:** cellulose acetate, nature products, composites, electrospinning, biocompatibility, bone tissue regeneration, osteoblast, osteoclast, cellular mechanism, signaling pathways

## Abstract

The specific objective of this study was to stabilize a simple valid method to prepare pure nanorod hydroxyapatite (HA) mixed with berberine chloride (BER) and Moghat water extract (ME) as composites for incorporation into cellulose acetate (CA) nanofibers to be used as novel bone scaffolds and to determine their efficacy in bone regeneration process In Vitro. Preparation of HA/BER and HA/ME composites were performed by mixing powders using the ball-milling machine. The HA, HA/BER, and HA/ME composites at a concentration of 6.25, 12.5, 25, 50, 100, and 200 mg were mixed with CA solution (13%), then the fiber was formed using electrospinning technique. The properties of the obtained CA fibers were investigated (SEM, TEM, EDX, FTIR, TGA, water uptake, porosity, and mechanical tests). The efficacy of HA and HA composites loaded into CA nanofiber on osteoblast and osteoclast differentiation were measured by tacking ALP, osteocalcin, TRAcP, calcium, and total protein concentration. Moreover, their effects on cell differentiation (CD90 and PARP- ɣ) and death markers (GSK3b, MAPK, Wnt-5 and β-catenin) were evaluated by using ELISA and qPCR. The obtained TEM results indicated that the continuous CA and CA/HA composites electrospun fibers have ultrafine fiber diameters of about 200 nm and uniform distribution of discrete n-HA clusters throughout. In addition, hydrocortisone (HCT) was found to increase the formation of adipocytes and osteoclastic markers CD90 and p38-MAPK which indicated the bone lose process take placed. Treatment with CA loaded with HA, HA/BER or HA/ME decreased CD90, Wnt-5, PARP- ɣ, GSK3b and p38-MAPK associated elevation of osteogenic markers: ALP and osteocalcin. Moreover, HCT overexpressed RANKL and down expressed Osterix gene. Treatment with CA/HA/BER or CA/HA/ME downregulated RANKL and upregulated Osterix associated with a reduction in RANKL/OPG ratio, at *p* < 0.05. In conclusion, novel CA composite nanofibers (CA/HA/BER and CA/HA/ME) reversed the HCT adverse effect on osteoblast cell death through canonical and non-canonical pathways regulated by Wnt/β-catenin and Wnt/Ca(^2+^) pathways. Furthermore, our data confirmed that the novel scaffolds create a crosstalk between RUNX-2, RANKL, p38-MAPK, and Wnt signals which positively impact bone regeneration process. Treatment with CA/HA/BER is better compared to the treatment with CA/HA/ME. Nevertheless, both are considered as alternative biomaterial scaffolds with a potential for biomedical applications in the field of bone tissue engineering.

## 1. Introduction

Tissue engineering is a vital area of regenerative medicine, with rapidly expanding research directed at preparing recent biomaterial matrices with adjusted properties to get functional tissues for specific applications [1]. Designing a biocompatible scaffold with optimal characteristics associated with the above is a major key element of successful tissue engineering strategies [2]. These developed scaffolds should have satisfactory biocompatibility and structure similar to that of the native extracellular matrix (ECM) in order to enable cells to grow and differentiate into specific tissues similar to their natural counterpart [3].

Bone tissues possess the intrinsic capacity for regeneration as part of the repair process in response to injury, as well as during skeletal development or continuous remodeling throughout adult life [4]. Bone healing is regeneration defined as a complex and well-orchestrated process of biological events of bone induction and conduction to optimize skeletal repair and re-store skeletal function [5]. However, in complex clinical conditions, bone regeneration is required in large quantities for the repair of large bone defects caused by trauma, infection, tumor resection, and skeletal abnormalities. To stimulate or augment bone regeneration of large bone defects, bone grafting such as autologous bone grafts and allografts is a commonly performed surgical procedure [6].

Several synthetic or nature in origin polymers such as polycarprolactone (PCL), poly(lactic-co-glycolic acid) (PLG), poly lactic acid (PLA), polyvinyl alcohol (PVA), collagen, gelatin, cellulose, chitosan, hyaluronic acid have been applied in bone tissue engineering applications [7]. The biodegradable polymers when implanted into a bone defect as a scaffold enhanced with signaling molecules to promote the bone formation process, cells differentiate into bone and are induced and expanded by the signaling molecules released from the polymer [7,8]. The cellulose-based scaffolds were attractive in biomedical and pharmaceutical applications because of their advantages regarding biocompatibility, high mechanical and thermal stability, non-toxicity, and also cost-efficiency [9]. The crystalline cellulose polymer is a naturally animal free occurring polysaccharide [10]. Cellulose is mainly produced by plants, although many bacteria, especially those belonging to the genus *Gluconacetobacter*, produce cellulose with mechanical and structural properties that can be exploited in numerous applications [11]. Cellulose consists of a linear chain of several hundred to over ten thousand β(1-4) linked D-glucose [12]. Cellulose is a diverse material as evidenced by a wide range of physical properties. The microfibril formation and crystallization can be adjusted by changing the cellulose source, solvent, and the preparation methods [13]. Cellulose-based scaffolds provoke an immune response as it reported that the non-toxic concentration of cellulose after the balance between type 1 T helper (Th1) and type 2 T helper (Th2) cells proliferation, therefore it increased the immunoglobulin production and consequently can be used for wound healing and inflammation because it exerts anti-inflammatory properties [14].

Cellulose acetate (CA) is an insoluble cellulose derivative. Cellulose acetate is partially acetylated cellulose, the acetyl content of which ranges from 29.0% to 44.8%, corresponding to mono-, di-, and triacetate [15]. CA shows great potential in single tissue engineering, mainly due to its biocompatibility and its ability to promote osteoblast proliferation and osteogenic cell differentiation. However, it has typically been used as a part of more complex composites, since CA itself does not have the necessary mechanical strength for load-bearing applications [16]. One of the most widely used biomaterials in bone tissue engineering is hydroxyapatite (HA), which has been applied in combination with CA to improve its biocompatibility, mineralization and mechanical properties.

The use of hydroxyapatite for bone tissue engineering is applied in several polymeric scaffolds and metallic implants [17,18]. Hydroxyapatite particles are used as additives for cellulose ester to boost the morphology and properties of the membranes [9,19]. Different forms of HA can be used for biomedical applications as porous and dense blocks, granules, paste, cement, nanorods, belts, and coatings. The nanorod-like HA is better than other forms because they not only reduce bone loss but also increase cortical bone thickness, and they mimic the natural bone HA nanocrystals (a plate-like shape with a length of 30–200 nm and a thickness of 2–7 nm) [18]. Nanorod hydroxyapatite enhanced osteoblast functions proliferation, alkaline phosphates, calcium synthesis, bone formation transcription factors production that correlate with decreased β-catenin accumulation leading to activation of the Wnt/β-catenin pathway [20]. The resorptive activity of osteoclasts cultured with nanorods HA was weaker than that of control osteoclastic cells. In addition, cell adhesion and proliferation were enhanced by the presence of HA. Therefore, nanorods hydroxyapatite may serve as a valuable scaffold for osteoporotic bone regeneration [20].

Berberine (BER) is an isoquinoline alkaloid that belongs to the structural class of protoberberines and is present in many plants including barberry. Berberine has been implicated in bone biology [21]. BER reduces osteoporosis by enhancing bone mineral density (BMD) and inhibiting osteoclast activity. In osteoblastic cells, BER enhanced the expression of osteogenic marker genes including osteopontin and osteocalcin, and promoted the transcriptional activity of the key osteogenic transcription factor Runx2 and RANKL [22]. Therefore, BER could also be a possible therapeutic agent to treat bone-related disorders including osteoporosis. Osteoporosis (OP) is a systemic skeletal disease characterized by a reduction in bone mineral density (BMD) and deterioration of skeletal microarchitecture, leading to increased bone fragility and fractures [23,24]. Samadian et al. 2020 implied that the incorporation of berberine into CA/Gelatin (CA/Gel) electrospun mat did not compromise the physical properties of dressing, while improving the biological activities [25].

Glossostemon bruguieri (Desf.), belongs to the family Sterculiaceae, and the plant grows wild in Iraq and Iran where it was imported. It was introduced to Egypt in 1932 and continued to be cultivated in a very small area [26,27]. The dried peeled roots of G. bruguieri (Desf.) (Known as Moghat in Arabic) are used in folk medicine as an anti-inflammatory, in autoimmune diseases, lupus, rheumatoid arthritis, and gout, to decrease the blood glucose level in diabetes, and as a tonic and nutritive agent. Furthermore, it was given to nursing mothers who need nutritive replenishment and for bone strengthening. From a previous study of Ghareeb et al., 2014, they reported that Moghat showed a positive effect on osteoporotic bone in the presence of valid Juvenile osteopenia (JO) experimental animal model confirmed by decreasing osteoclastic resorption and increasing osteoblastic formation markers. Their results strongly suggest that Moghat might be a potential therapy for the management of JO in humans, as it combines a powerful bone-forming as well as an anti-resorptive activity [26].

These components (HA, BER, and Moghat) when used separately provide a relatively successful mean of augmenting bone growth, nonetheless, the composite of these materials will exceed this success. The combination of HA with natural products has also been developed and studied in purpose to improve the properties of HA as biomaterial or vice versa improve the therapeutic effect of this natural product. Finding new bone grafts or improving the available grafts in the market will help patients, decreases the bone morbidity rate, and increases the investment because we will be able to produce cheaply and locally producing bone grafts for bone tissue regeneration.

Electrospinning is a simple, accessible, and efficient technique for the production of nanofibrous mat of various materials [28]. This method can be used to improve the scaffold structure, porosity, surface, and alignment [29]. Electrospinning is of interest technology platform for the design and fabrication of nanofiber-based scaffolds to mimic the hierarchical architecture of the extracellular matrix (ECM) [30], and manipulate cell behaviors thereby favoring the infiltration and viability of cells [29]. This technique is a solution-based approach that relies on the electrostatic repulsion between surface charges to form nanofibers. In electrospinning, nanofibers are formed when a solution of a viscoelastic polymer is extruded through a stainless-steel needle at high voltage. This method has been applied to prepare biomimetic and basic scaffolds from a variety of natural and synthetic biomaterials [29]. This method is utilized to fabricate nanofibers of polymers, metals, ceramics, and composites. Nanoparticles are mixed with polymers and electrospun to produce biomimetic and basic scaffolds.

The aim of this work was to design new bone scaffolds. Nanorod hydroxyapatite (HA) and HA composites (HA/BER and HA/Moghat extract (ME)) were incorporated into cellulose acetate fiber by electrospinning technique to obtain porous scaffolds used in different medical applications. The CA different scaffolds are characterized by a combination of physical, chemical and biological properties. The presence of HA, HA/BER, and HA/ME in the CA polymer mat is advantageous for osteoblast proliferation and improves the surface mineralization process in vitro. This is achieved by osteoporosis induced by cortisone in osteoblastic cells isolated from rat long bones through crosstalk between different signaling pathways as Wnt/β-catenin signaling, Runx2 signaling, and p38-MAPK signaling pathways.

## 2. Materials and Methods

### 2.1. Materials

Cellulose acetate (CA; white powder; Mw ≈ 30,000 Da; acetyl content = 39.7 wt.%; degree of acetyl substitution ≈ 2.4) was purchased from Sigma Aldrich (Schaffhausen, Switzerland). Berberine chloride hydrate (BER; C_20_H_18_ClNO_4_, >98% purity) was purchased from Sigma Aldrich (Darmstadt, Germany). Acetone (CH_3_COCH_3_), Dimethyl sulfoxide (DMSO), and 3-(4, 5-dimethylthiazol-2-yl)-2, 5-diphenyl tetrazolium bromide (MTT) were obtained from BIO BASIC, (Markham, Canada). Minimum essential medium Eagle—alpha modification (α-MEM), Dulbecco’s modified Eagle’s medium, with 4.5 g/L glucose and L–glutamine (DMEM), L-glutamine, Penicillin 10,000 IU/mL and streptomycin 10,000 μg/mL, and Trypsin-EDTA solution containing 0.25% trypsin was manufactured by Lonza (Verviers, Belgium). Fetal bovine serum (FBS, heat inactivated) was purchased from EuroClone SpA (Milan, Italy). Apigenin (5,7-dihydroxy-2-(4-hydroxyphenyl) chromen-4-one, C15H10O_5_, 1 mg/mL) was purchased from Merck KGaA (Darmstadt, Germany).

### 2.2. Plant Material

The air-dried peeled roots of *G. bruguieri* (Desf.), family Buttneriaceae (Sterculaceae), were purchased from Abu Madi Egyptian local market in Alexandria, Egypt. The plant material was authenticated by Prof. Dr. Eldareir, S. Prof. of plant ecology, Faculty of Science, University of Alexandria, Egypt.

### 2.3. Animal and Ethical Approval

Male neonatal Wister albino rats (10 ± 5 g, aged 0 to 5 days) were obtained from Experimental Animal House, Institute of Graduate Studies and Research—IGSR, Alexandria University. All procedures for animals were performed at Animal House of Pharmaceutical and Fermentation Industry Development Center (PFIDC), City of Scientific Research & Technological Applications (SRTA-city), New Borg El Arab, Alexandria. All experimental designs were approved from PFIDC Institutional Animal Care and Use Committees (IACUSs) where the approval number was IACUC#45-13M-1021.

### 2.4. Preparation of HA/BER and HA/ME Composites Loaded CA Fiber

#### 2.4.1. Preparation of Moghat Extract

Moghat roots were ground into powder form in a Jaw Crusher (BB 50 Retsch, Germany), then 25 g of powdered roots were soaked in 500 mL boiled sterilized distilled H_2_O for one day at 37 °C in shaker incubator (350 r.p.m.) [26,27]. The suspension was collected and filtrated using 24.0 cm filter papers. The filtrate was concentrated by evaporation at 60 °C under vacuum to sticky solution. The resident sticky solution was lyophilized and weighed. The lyophilized powder was ball-milled in High Energy Ball Mill (Emax, Retsch, GmbH, Haan, Germany) for 30 min at 300 r.p.m and ball to powder ratio was 10:1 (w:w) to obtain fine and homogenate powder then stored in labeled containers (ME) at 4 °C until used.

#### 2.4.2. Preparation of HA/BER and HA/ME Composites

Hydroxyapatite nanorod powder was synthesized from our previous reported work Shaban, N.Z. et al. 2021 [31] by microwave-irradiation method via a home model microwave. Using ortho-phosphoric acid H_3_PO_4_ (0.1 M) solution and calcium nitrate tetrahydrate Ca(NO_3_)_2_.4H_2_O (0.167 M) solution as sources of phosphorous and calcium, respectively. The synthesized nanorod HA powder was separately mixed with BER and ME powders in High Energy Ball Mill (Emax, Retsch GmbH, Haan, Germany) for 30 min at 300 r.p.m and ball to powder ratio was 10:1 (w:w) to obtain a fine and homogenate nano–rod HA/BER and HA/ME composites powder then stored in separate labeled containers until used.

#### 2.4.3. Preparation of HA, HA/BER, and HA/ME Composites-Loaded Cellulose Acetate Fiber

The Cellulose acetate (CA) powder (10–16 g) was dissolved in 100 mL acetone solvent at different concentrations of 10–16% (*w*/*v*). Then, 6.25, 12.5, 25, 50, 100, 200 mg of the HA powder, HA/BER, and HA/ME composites were separately suspended into 10 mL of CA solution at a concentration of 13% (*w*/*v*). The mixed solutions were stirred at room temperature for 24 h and ultrasonicated for 15 min. Prior to e-spinning, the as-prepared solutions were characterized for their viscosity at RH6 Spindle, 100 r.p.m and 25 °C using a digital rotational viscometer (ViscoQC 100, Anton Paar, Sydney, Austria) [32]. Preparation of all fiber samples was carried out at 25 °C.

The prepared solutions were transferred into a 5 mL syringe, with 0.8 mm needle gauge. The electrospinning was conducted using electrospinning unit (NanoNC- ESR100D, Seoul, Korea). These mixtures were then e-spun under controlled electric field of 24–30 kV applied to the needle. The distance between the needle tip and aluminum collector was adjusted at 15 cm. All solutions were ejected at a fixed feeding rate of 0.5 mL/h using syringe pump (NanoNC-EP100D, Seoul, Korea). All samples were synthesized at our laboratory using reported procedures [33,34,35]. The optimization of the e-spinning conditions is demonstrated at Table 1. The resulting fiber was dried overnight prior to peel-out from aluminum foil to remove any solvent left on its surface. The collection time was w24 h to obtain uniform mats with a thickness of 200–250 µm and diameter of 200 ± 20 mm. The thickness of mat was assessed by a digital micrometer (Mitutoyo- IP65, Neuss, Germany).

### 2.5. Physical Characterization of Scaffolds Samples

For morphological appearance of all the fibers samples (circular disc; w5 mm in diameter) were sputter coated with gold and investigated microscopically using a scanning electron microscope (SEM, JEOL JSM 6360LA, Tokyo, Japan). After SEM characterization results, we select the best fibers samples CA13, CA13/HA50, CA13/HA/BER100, and CA13/HA/ME100 to complete the characterization and biological application. Microscopic size of the four selected fibers sample were characterized by transmission electron microscopy (TEM, JEOL 2100 PLUS, Tokyo, Japan) along with electron dispersive X-ray (EDX, Gatan, Pleasanton, CA, USA). Thermal stability of the four fibers samples was studied using Thermo gravimetric analyser (TGA-50 Shimadzu, Tokyo, Japan) the temperature program was raised from room temperature to 800 °C at a heating rate 10 °C/min under nitrogen flow rate 20 mL/min, Fourier-transform infrared spectroscopy (FTIR-8400 S Shimadzu, Tokyo, Japan) was used to detect the distinguished functional groups of the prepared samples. The recorded region is from 400–4700 cm^−1^ using the spectroscopic grade potassium bromide (KBr) pellet technique.

### 2.6. The Mechanical Testing of the Scaffolds

The samples were cut according to American Society for Testing and Materials (ASTM D882) for sheeting having a nominal thickness no greater than 0.25 mm (0.010 in.) [36]. Samples were processed into rectangular strips of length = 50 mm, width = 10 mm, and thickness around 0.2 mm. To determine the surface roughness values of fibers samples, a portable surface roughness tester (Mitutoyo Surftest, SJ-201, Kawasaki, Japan) was used. It is a hand-held electronic instrument that measures the peak-to-valley height of the surface profile of cleaned surfaces. Three samples were measured for each fibers samples (CA13, CA13/HA50, CA13/HA/BER100, and CA13/HA/ME100) to obtain the mean roughness value [37]. After the surface roughness testing was completed, the same samples were used to determine the tensile strength and percent elongation at break values of the fibers were determined at room temperature using universal testing machine (EZ (5 KN), Shimadzu, Tokyo, Japan), with a crosshead speed 1 mm/min [36]. Each measurement was replicated three times per sample.

### 2.7. Water Uptake, Porosity and Contact Angles of the Scaffolds

Water-uptake capacity which shows the capacity of the fibers to absorb culture media or body fluids and the weight loss behavior of both the neat and the drug-loaded e-spun CA fiber mats were measured in phosphate buffered saline (PBS) (0.01 M, pH 7.4) at the physiological temperature of 37 °C for 24 h. The CA13, CA13/HA50, CA13/HA/BER100, and CA13/HA/ME100 composite bandages were cut into small pieces having equal weights and immersed in PBS for water uptake study. The bandages were taken out after 24 h and the PBS adhered on the surface was gently removed by blotting with filter paper and weighed immediately (W1) [38]. The water uptake was calculated using the following equation:

Water uptake (%) = [(W1 − W0)/W0] × 100(1)

where W0 is the weight of dry samples and W1 is the weight of samples after immersion in PBS buffer at room temperature for 24 h. The experiment was repeated three times to determine the standard deviation.

The porosity assessment of the scaffold was determined by a modified liquid displacement method for small size scaffolds [25,39]. The total porosity is related to the amount of pore space present in the scaffolds. The scaffold was immersed in a 5 mL measuring barrel containing ethanol for 10 min until the air overflowed completely. The porosity is calculated as follows:

Porosity (%) = [(M2 − M3 − M_S_)/(M1 − M3) × 100 (2)

where M1 is the initial mass of the bottle filled with ethanol, Ms is the mass of the dry scaffold, M2 is the mass of the scaffold submerged in absolute ethanol inside the bottle, and M3 is the mass of the bottle after the scaffold was gently removed.

The water contact angle and surface energy Measurements of the water contact angle were recorded at room temperature using a Contact angle meter VCA 2500 XE equipped with charge-coupled device (CCD) camera and analysis software (AST Products, Billerica, MA, USA) to determine the contact angle of water droplet with the various CA13 loaded fibers as an indication for their hydrophilicity [40,41]. Prior to measurement, a 0.25 mm thick layer of fiber was applied to the glass in order to obtain a relatively flat surface. Static contact angles were measured 10 s after placing 3 µL of distilled and deionized water on the sample surfaces. All experiments were conducted in ambient conditions and were performed at least in triplicate per sample. Surface energy (E surface) was calculated using the equation Neumann equation of the state’s approach:

E surface = Elv × cos θ(3)

where Elv = 72.8 mJ/m^2^ at 20 °C for pure water and θ is the measured contact angle.

### 2.8. Release of Drugs (BER and ME) from HA Composites-Loaded CA13 Fibers

Nanofibrous mats were cut into 1 cm × 1 cm pieces and weighted (≈5 mg) to define total mass of model drugs (HA/BER 100 mg/10 mL CA13 and HA/ME 100 mg/10 mL CA13) present in the CA13 mats and then placed in vials filled with 10 mL of PBS solution (0.01 M, pH 7.4). The aim of maintaining material in 37 °C of PBS buffer was to imitate conditions of the human body. After selected time intervals, materials were placed in a fresh PBS solution and constantly stirred at a speed of 100 rpm. At particular time interval, 1 mL samples were taken, and an equal volume of fresh PBS was immediately added after each sampling [42,43]. All samples were protected from light to avoid photo bleaching.

For quantitative assessment of BER release, a calibration curve of standard berberine chloride at a known concentration (2–10 mg/mL) was measured using the UV–vis spectrofluorometer at the wavelength of 415 nm against the blank (distilled water) [42,44]. The amount of released moghat extract (ME) was indirectly determined via high-performance liquid chromatography (HPLC) (Shimadzu, Tokyo, Japan) using the Apigenin (5,7-dihydroxy-2-(4-hydroxyphenyl)chromen-4-one, C_15_H_10_O_5_) (1 mg/mL) as a standard. The mobile phase was prepared by mixing acetonitrile with 0.1% acetic acid in (45:55, V/V). The UV detector was set at 268 nm [27,45].

### 2.9. Isolation of Osteoblast Cells and Induction of Osteoclast Cells Formation

Rat calvarial osteoblast (RCO) cultures were used as a model to assess osteoblast behavior and differentiation. Cells were isolated from 0 to 5 days old neonatal male Wister albino rats according to Shaban, N.Z. et al. 2021 our previous study [20]. The parietal and occipital bones were dissected and washed with phosphate buffered saline (PBS). Harvest bones were mincing and subsequently digested by incubating in 700 units/mL of type 1 collagenase at 37 °C. The supernatant of the first digestion was discarding and the calvarial fragments were treated five times with collagenase (20 min at 37 °C) and the subsequent supernatants were collected, combined and sediment. The resulting cell pellet was resuspended and cultured in cultured media (CM) α-MEM supplemented with 10% (\emph{v}/\emph{v}) fetal bovine serum (FBS), and antibiotics (200 U/mL penicillin and 200 µg/mL streptomycin). RCO cultures were maintained in a humidified 5% CO_2_ atmosphere at 37 °C.

Induction of osteoporosis using glucocorticoids was carried out according to the protocols of our previous study Shaban, N.Z. et al. 2021. Osteoblastic cells were plated into six-well sterile culture plates at 10^5^ cells/well and incubated in CM until a confluent layer was achieved. Then, 0.5 mg/mL hydrocortisone (HCT) was added and every 3–4 days the media was changed. After 21 days of culture, osteoclast-like cells formation were observed [20].

### 2.10. Cell Viability and Proliferation Test

The selected fibers samples: CA13, CA13/HA50, CA13/HA/BER100, and CA13/HA/ME100 (circular disc; w 5 mm in diameter) were plated in 96-well cell culture plate and exposed to a UV lamp for 1 h in both scaffold sides. Then, 200 μL of CM were added to the scaffolds for 24 h to insure complete sterilization [46].

Subsequently, the 200 μL of cultured medium was removed and a fresh CM contained 1 × 10^5^ osteoblast or osteoclast/well were added followed by the plate incubation for 7 days in CO_2_ incubator (37 °C, 5% CO_2_, and 90% relative humidity). At the end of treatment period, MTT was firstly prepared as a stock solution of 5 mg/mL in phosphate buffer saline PBS (pH 7.2) and filtered. Then, the addition of 20 μL of MTT solution for each well was done and then plate was incubated for 4 h in a CO_2_ incubator. After incubation, the plate was centrifuged at 1650 rpm for 10 min and the medium was discarded. The formazan crystals (MTT byproduct) were re-suspended in 100 μL DMSO and the reading was measured at a wavelength of 570 nm [47].

The % viability was calculated as follow: (AT/AC) × 100(4)

AT = mean absorbance of cells treated with different HA samples.

AC = mean absorbance of control untreated cells with culture medium only.

### 2.11. Culture of Osteoblast (OB) and Osteoclast (OC) on the Selected Fibers Samples

The prepared scaffolds (circular disc; w 10 mm in diameter) were sterilized as mentioned before, but they were plated in 24-well cell culture plate with 500 μL of cultured medium. The osteoblast or osteoclast cells at a concentration of 1 × 10^5^ cells/well were allowed to groin complete media (10% FBS, 2 mM L-glutamine in α-MEM) for 2 days to reach 70% confluence then collected fresh media. Thereafter, CM media were removed from the scaffolds wells and replaced with fresh medium containing osteoblast or osteoclast cells. Cells were incubated with the scaffolds at 37 °C, 5% CO_2_ for 7 days. The adherent cells were enzymatically detached with trypsin/EDTA solution (1×) and then the suspended cells collected by centrifugation at 400× *g* for 5 min. The cell pellets were washed three times with phosphate buffer saline (PBS). Then, cells were resuspended in PBS (pH 7.4) containing protease inhibitor and subjected to ultrasonication for 3 times. The supernatant was collected for quantification of biochemical parameters.

### 2.12. Bone Remodelling Biomarkers

Proliferation markers such as osteocalcin (OCN), alkaline phosphatase (ALP), and extracellular calcium concentration were assayed using commercial kits supplied from Nordic Bioscience Diagnostics (Herlev, Denmark), Biosystems (Barcelona, Spain), and Sigma-Aldrich (St. Louis, MO, USA), respectively, while total protein concentration was assayed according to Biosystems (Barcelona, Spain) and Tartrate-resistant acid phosphatase (TRAcP) as a marker of bone resorption was assayed according to Takara Bio Inc (Kusatsu, Japan).

### 2.13. Molecular Investigations

Total RNA was isolated from the cells using easy red^TM^ total RNA extraction kit (iNtRON Biotechnology, Seongnam, Korea) according to the manufacturer’s protocol and quantified spectrophotometrically. One µg of total RNA was reverse transcribed using Maxime RT PreMix kit (iNtRON Biotechnology, Seongnam, Korea) in accordance with the manufacturer’s protocol. The qRT-PCR analysis was performed as follow: 1 µL of cDNA was added to specific primers (10 µM) shown in (Table 2), RealMOD™ Green ^w2^ 2× qPCR mix, 10 µL (iNtRON Biotechnology, Seongnam, Korea) and RNase-free water in a total volume of 20 µL per well. The qPCR amplification step was performed with 35–40 cycles of 95 °C for 20 s, 43–60 °C for 30 s and 72 °C for 60 s shown in Table 2. The qRT-PCR was performed using Roto Gene Q 5Plex HRM (Qiagen, Hilden, Germany) with dedicated Rotor-Gene Q software. Samples were loaded in duplicate. Data were calculated using the comparative 2^−ΔΔCT^ method, and all values were normalized to the mRNA level of glyceraldehyde-3-phosphate dehydrogenase (GAPDH) gene. The results were expressed as n-fold increase in gene expression using calibrator results of sham control. The primer sequences of targeted genes and qRT-PCR conditions are summarized in Table 2.

Levels of Wnt-5, β-catenin, Glycogen synthase kinase 3 beta (GSK3β) and the phosphorylation of GSK3β at pS9 (GSK3β pS9) were measured by manual quantitative ELISA technique using rabbit polyclonal Wnt-5 (# PA5-35344, ThermoFisher, Waltham, MA, USA), rabbit monoclonal β-catenin and GSK3β (#8480 and #9315, respectively, Cell Signaling Technology, Danvers, MA, USA) as well as rabbit polyclonal GSK3β pS9 (#abx328236, Abbexa, Shanghai, China). Antigen to a final concentration 100 µg protein was diluted in a coating buffer (bicarbonate/carbonate, 0.2 M, pH 9.6) in the wells of a polyvinyl chloride (PVC) microtiter antibodies were diluted in blocking solution. After incubation with primary and secondary antibody (Goat anti rabbit IgG, ALP, #A8025, Sigma-Aldrich, St. Louis, MO, USA), substrate solution (p-nitrophenyl phosphate, disodium salt, PNPP) was added. The reaction was stopped using sodium hydroxide (3 M) and the color developed was read at 450 nm on a plate reader (Sanofi Diagnostics Pasteur, Lyon, France). Standard curves for each protein were constructed [48]. GSK3β and GSK3β pS9 concentration were expressed in µg/mg protein while Wnt-5 concentrations were expressed in ng/mg protein, and β-catenin concentrations were expressed in pg/mg protein.

Determination of CD90, Poly (ADP-ribose) polymerase (PARP-ɣ) and p38-mitogen-activated protein kinase (p38-MAPK) proteins levels were determined by using ELISA kit according to manufactures instructions. The concentration of p38 MAPK, CD90 was expressed in ng/mg protein and PARPɣ concentration were expressed in pg/mg protein.

### 2.14. Statistical Analysis

Statistical Product and Service Solutions (SPSS) software package version 20.0 (IBM Corp, Armonk, NY, USA) was used for data analyses. Data are presented as mean and standard deviation, significance among samples at *p* < 0.05 was assessed by using one-way analysis of variance (ANOVA).

## 3. Results and Discussion

**Figure 1:**
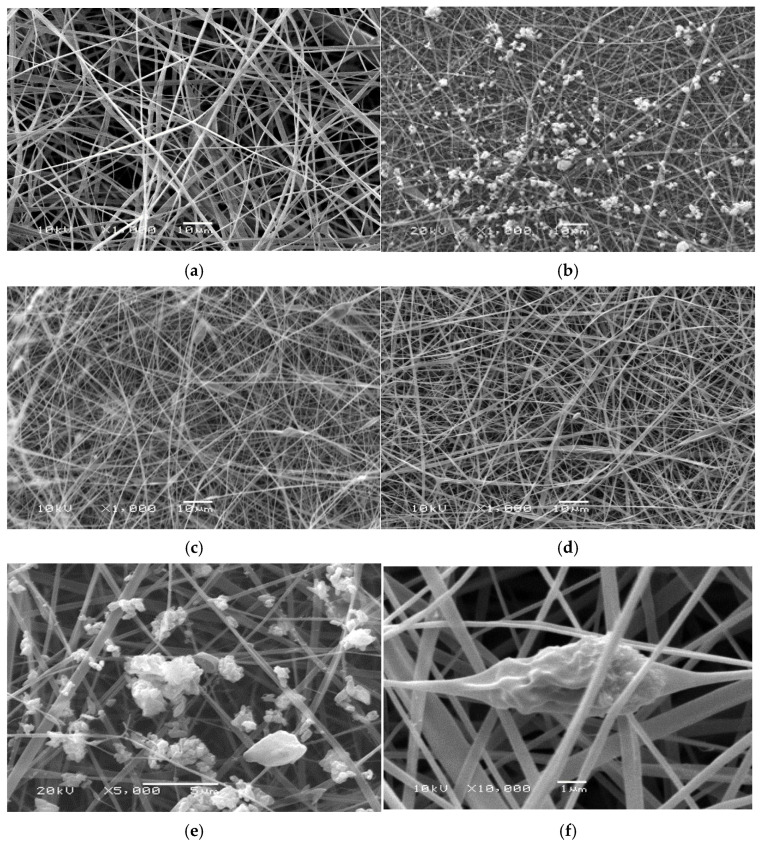
Scanning electron microscopy of fibers: (**a**) CA13 fiber and (**b**) n-HA particles in CA13 fiber (CA13/HA), (**c**) nHA/BER composites in CA13 fiber (CA13/HA/BER), (**d**) nHA/ME composites in CA13 fiber (CA13/HA/ME). (**e**,**f**) Scanning electron microscopy of HA particles dispersed inside CA13 fibers at different fields and at different magnifications (5000× and 10,000×).

**Figure 2:**
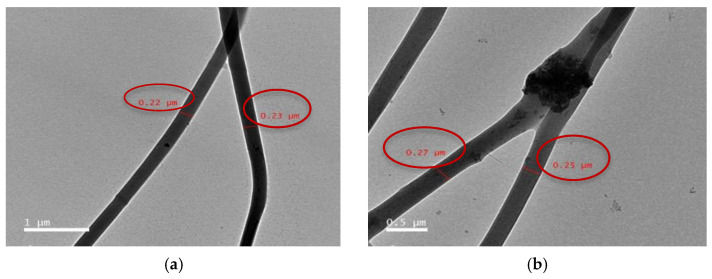
Transmission electron microscope of prepared fibers: (**a**): CA13 fiber at image scale 1 µm. (**b**): HA particles dispersed inside CA13 fibers at Images scale 0.5 µm.

All SEM images for the prepared fibers were shown in the supplementary material file (Figures S1–S4). Selected SEM images of the e-spun fibers from these solutions are shown in Figure 1. Clearly, cross-sectional round fibers were obtained and there was no presence of any kind of HA or HA composites aggregates on the surface of these fibers, implying that the as-loaded HA, HA/BER, and HA/ME were perfectly incorporated well within the CA13 fibers. SEM results indicate the formation of uniform non-woven nanofiber continuous CA13 fibers, and the surface of CA13 nanofibers was observed to be smooth and regular (Figure 1a). While for CA13/HA50, CA13/HA/BER100, and CA13/HA/ME100, surface roughness and irregularities are observed, due to the presence of the HA, HA/BER, and HA/ME composites loaded on the surface and inside the nanofiber mats (Figure 1). The presence of the HA on the nanofiber mats was confirmed by the SEM and TEM analysis (Figure 1) and (Figure 2) respectively. The results demonstrated HA rod-like crystals aggregate all through the surface and inside the fiber mats. The size of the fibers was calculated according to the Image J software and varies within 230 nm.

**Figure 3:**
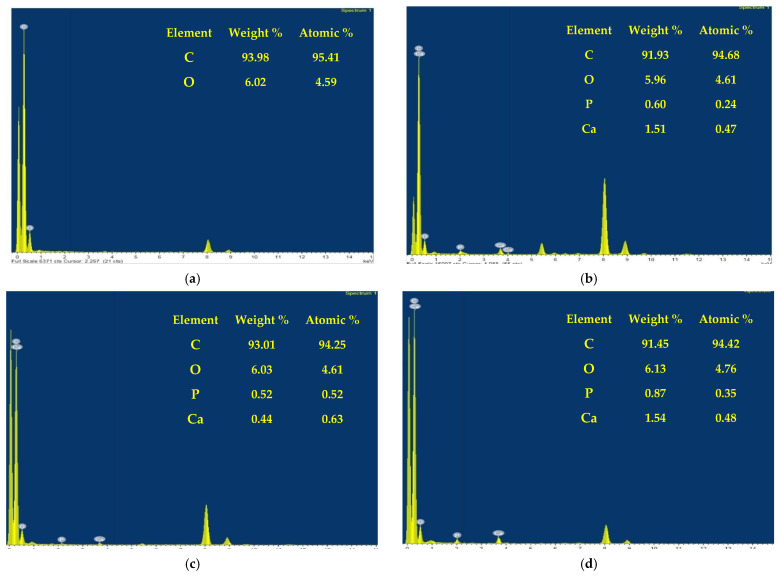
TEM coupled with EDX results of the nanofibers before and after HA/NPs composites loading in CA13 mats. (**a**) represents EDX results of CA13 nanofibers; (**b**–**d**) represent the EDX data of the loaded CA13 nanofibers containing HA, HA/BER, and HA/ME, respectively.

TEM along with EDX results of the nanofibers before and after HA/NPs composites loading of CA13 mats represented in Figure 3: In this figure, we can observe the pristine CA13 nanofibers, which are free of HA, do not show any significant peak indicating the presence of Ca and P (Figure 3a). In contrast, the nanofibers modified with HA, HA/BER, and HA/ME indicate the presence of both Ca and P peaks, further enriching the hypothesis about the beneficial attribute displayed by the presence of HA and HA composites in the CA13 nanofibers. Furthermore, the stoichiometric Ca/P Ratio is ~1.67. However, the concentration of Ca and P after loading on CA13 fibers is independent of the ratio of original loading of HA in the cellulose acetate solutions that were used in the electrospinning process.

**Figure 4:**
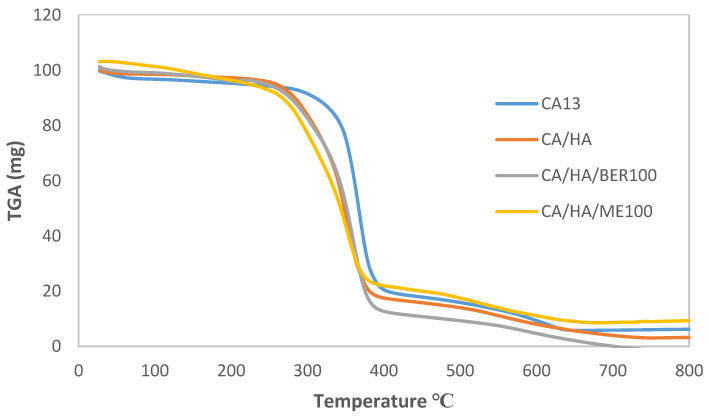
Thermal analysis pattern of different CA13 scaffolds. Data analysis by TGA device software.

Thermogravimetric analysis (TGA) was carried out to obtain the weight loss (degradation profiles) of a material and its thermal stability. Figure 4 shows the TGA analysis for pure CA nanofiber (CA13), and different CA composite nanofiber (CA13/HA, CA13/HA/BER, and CA13/HA/ME). All CA scaffolds demonstrated quite close TGA profiles as indicated in Figure 4. The results confirm that introducing HA, HA/BER, and HA/ME composites to the CA polymer leads to a slight shifting of the thermal decomposition of CA to a lower temperature. In general, all prepared scaffolds were thermally stable below 250 °C; therefore, all CA13 scaffolds were suitable for pharmaceutical and biomedical processing.

**Figure 5:**
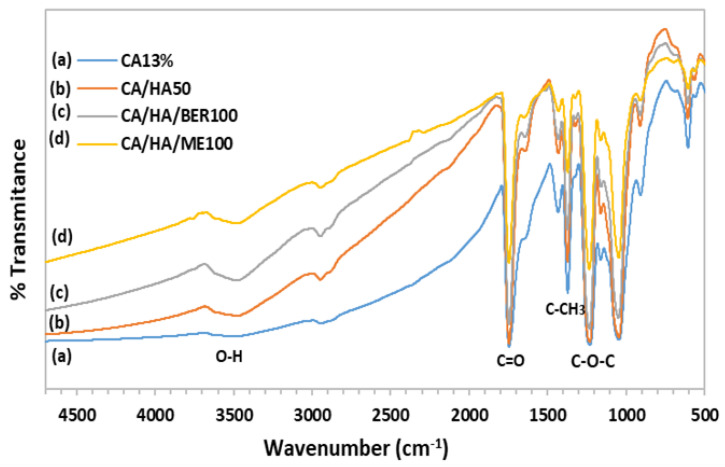
FTIR graphs for different CA13 scaffolds. (**a**) CA13% (**b**) CA13/HA50 (**c**) CA13/HA/BER100 and (**d**) CA13/HA/ME100.

Figure 5 shows the FTIR spectrum of pure CA13 nanofiber and CA/HA50, CA13/HA/BER100, and CA13/HA/ME100 composites nanofibers. Pure CA nanofiber demonstrated in (Figure 5a) a characteristic absorption band of C=O stretching at 1744 and 1228 cm^−1^ was attributed to acetate substituent presented by C–O–C alkoxyl stretching. In addition, the band at 3480 cm^−1^ was attributed to O–H stretching vibration, whereas the 1370 cm^−1^ was attributed to C–CH_3_ methyl bending [3]. Figure 5b–d shows the characteristic peaks for both CA and HA50, HA/BER100, and HA/ME100. From these results, we can conclude that all CA incorporated with HA pure powder or HA composites were well blended, interfered and formed smooth composites fiber mats during the e-spinning process.

The results of shear viscosity are summarized in Table 3. The results showed that the initial shear viscosity of the CA13 solution with acetone was the highest viscosity value 2.399 ± 0.32. While, the presence of HA, HA/BER and HA/ME in the base CA13 solution was responsible for the observed decrease in the shear viscosity of the e-spinning solution. This may be due to the decrease in the aggregation in the CA polymer chain structure by the incorporation of HA and HA different composites [25].

The net CA fiber exhibited low tensile strength and high elongation at break compared with the CA mats incorporated with HA or HA composites indicate the stiffer of the net CA material. The increase in tensile strength can be attributed to interaction between the CA fibers after the incorporation. As a result, the mechanical strength of incorporated CA novel scaffolds material was dramatically improved.

Contact angle data, surface characteristic and water uptake of CA13 fibers before and after loading with HA, HA/BER, HA/ME nanocomposites: The cellulose acetate membrane had a hydrophobic contact angle of 113.46°. Loading of HA/NPs composites changes the permeability of the membrane, the CA13/HA50 and CA13/HA/BER100 composites membranes demonstrated slight hydrophilicity with a measured contact angle of 53.46 and 56.46° is probably due to the increased the porosity after addition of drugs. While, in the case of CA13/HA/ME100, a relatively higher contact angle of 103.46° was observed. Surface energy calculations show that there is an increase in surface activity after being loaded with the HA/NPs composites compared to pure CA13 membranes. The results of our study show that loading of CA13 mats with HA/BER and HA/ME composites has a direct effect on water-uptake capacity of the nanofibrous mat. Addition of composites enhanced water uptake value (Table 4). The differences between water uptake values are not statistically significant.

As shown in Table 4, the porosities of Scaffolds CA13, CA13/HA50, CA13/HA/BER100, and CA13/HA/ME100 were determined to be 0.89 ± 0.04, 0.98 ± 0.51, 0.99 ± 0.23, and 1.01 ± 0.67, respectively. Whereas the porosities of CA13/HA, CA13/HA/BER, and CA13/HA/ME are not significantly different, the porosity of CA13 was significantly lower in comparison. This indicates that the three kinds of scaffolds are highly porous and the porosity increase with the incorporation of HA, HA/BER, and HA/ME into the CA13 scaffold. Chong et al. concluded that the porosity in the range of 60–90% is preferred for tissue engineering applications [25].

**Figure 6:**
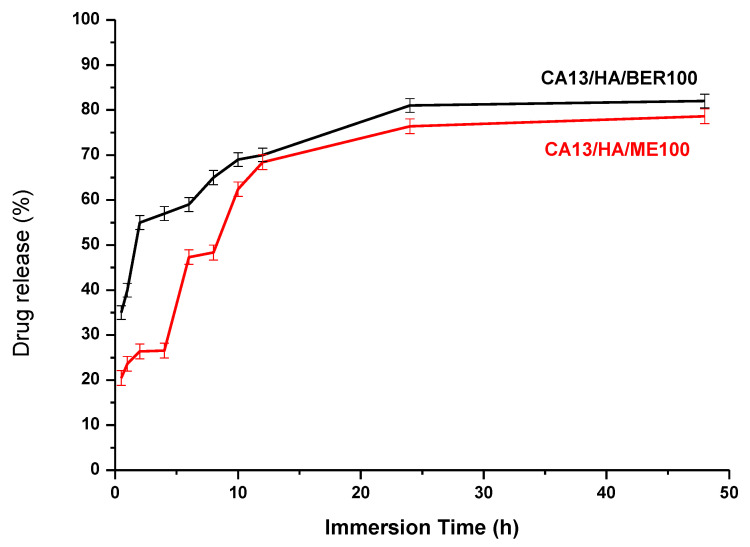
The in vitro release profile of Berberine and Moghat from CA13 Electrospun Fiber Mat in Phosphate Buffer Saline (pH 7.4) at body Temperature (37 °C).

In vitro drug release: release characteristics were carried out in phosphate buffer saline. The amount of berberine and moghat released from the drug-loaded fibers is demonstrated in Figure 6. The total drug content was determined prior to the release studies to calculate the release percentage of the actual composites content of each fiber matrix. Berberine or Moghat release from CA13 fibers was an initial low release, followed by a degradation-dependent release from the fibers.

Table 5 shows that treatment of osteoblast (OB) cells with CA13/HA/BER100, CA13/HA/ME100, and CA13/HA50 scaffolds was significantly increased TRAcP activities than that of control (OB) cells. On the case of osteoclast (OC) cells, the addition of CA13 different fibers significantly decreased TRAcP levels comparing to normal OC cells. The lowest level was shown in case of CA13/HA50 fiber mats treatment while the highest level was shown in case of CA13/HA/BER treatment at *p* > 0.05. The treatment of OB cells with CA 13, CA13/HA/BER100 and CA/HA50 was significantly increased calcium level than that of control (OB) cells. In the case of OC cells, addition of different fibers significantly increased calcium levels in the same proportion compared to normal OC cells at *p* > 0.05. The treatment of OB cells with CA 13 and CA13/HA/BER100 significantly decreased total protein level than that of control (OB) cells. The CA13/HA50 treatment increased total protein level when compared to control OB cells. In the case of OC cells, the addition of different fibers significantly decreased the total protein levels comparing to normal OC cells. The lowest level was showed in the case of CA13/HA50 fiber treatment at *p* > 0.05. The treatment of OB cells with CA13 was significantly decreased the cell viability than that of control (OB) cells. While, the treatment with CA13/HA/BER100 and CA/HA/ME100 did not affect the cell viability compared to that of control (OB) cells. In contrast, the CA13/HA50 treatment showed the highest level of cell viability at *p* > 0.05. In the case of OC cells, the addition of different fibers significantly increased cell viability compared to normal OC cells. The lowest level was shown in case of CA13/HA50 disc treatment while the highest level was shown in case of CA13/HA/BER100 and CA13/HA/ME100 treatment at *p* > 0.05.

Table 6 demonstrates that HCT increased the mesenchymal stem cells (MSC) markers CD90 and p38-mitogen-activated protein kinase (p38-MAPK) as well as PARP- ɣ. MSC is preadipocytes that subsequently differentiate into mature adipocytes. Therefore, HCT increased the formation of adipocytes in bone tissue that contribute to osteoclast formation. The treatment with HA/BER100 or HA/ME100 carried on CA decreased CD90, p38-MAPK and PARP- ɣ that associated with elevation of osteogenic markers; ALP and osteocalcin, which indicated that the differentiation of MSC into osteoblast or the prevention of osteoclast formation.

Table 7 showed that the addition of HCT to the media increased the cellular Glycogen synthase kinase 3b (GSK3b) and Wnt-5. Activation of this pathway induces osteoblasts cell death. Despite the direct HCT effect on Wnt/β-catenin pathway, inflammation microenvironment induced β-catenin accumulation which increased also when adipocytes number was increased in order to suppresses the non-canonical Wnt/Ca(^2+^) pathway thus leading to cell proliferation positive increment that associated with osteogenic differentiation reduction [20]. Treatment with HA/BER or HA/ME carried on CA13 fiber improved the tested parameters but did not normalize them. In conclusion, both treatments reversed the HCT adverse effect on osteoblast cells death through canonical and non-canonical pathways which is regulated by Wnt/β-catenin and Wnt/Ca(^2+^) pathways. Statistically, the treatment with CA13/HA/BER is better than treatment with CA13/HA/ME, at *p* < 0.05.

Table 8 indicates that HCT decreased the markers of bone proliferation: Osterix, Runx-2 and collagen a1 (Cola1) while treatment with CA13/HA/BER or CA13/HA/ME increased the osteogenic markers. Furthermore, treatment with CA13/HA/BER nanofiber was better than treatment with CA13/HA/ME, at *p* < 0.05.

Table 9 shows that HCT increased the markers of bone resorption: sclerostin (SOST), Dickkopf-1 (DKK), and transcription factor SOX9. On the contrary, treatment with HA/BER or HA/ME carried on CA13 decreased the markers of bone resorption and treatment with HA/ME was better than treatment with HA/BER, at *p* < 0.05.

Finally, Table 10 demonstrates that HCT increased the receptor activator of nuclear factor kappa beta (RANKL) gene expression and downregulated Osterix expression, all these markers control Wnt/catenin pathway and inflammatory pathway. Treatment with CA13/HA/BER or CA13/HA/ME downregulated the receptor activator of nuclear factor kappa beta (RANKL) and upregulated Osterix associated with the reduction in RANKL/osteoprotegerin (OPG) ratio, at *p* < 0.05. Treatment with CA13/HA/BER was better than treatment with CA13/HA/ME, at *p* < 0.05. The new scaffolds caused a crosstalk between Runx-2, p38-MAPK, and Wnt signal pathways and they had a positive impact on bone regeneration.

## 4. Conclusions

Cellulose Acetate fibrous mats were fabricated through the electrospinning process. The addition of BER or ME to uniform non-woven cellulose acetate nanofiber converted it to irregular-surface rough nanofiber. The drug release profile proved that berberine release from CA13 fibers was an initial low release, followed by degradation-dependent release from the fiber. Finally, the prepared CA13 incorporated with HA/BER100 and HA/ME100 composites nanofibers are stable and suitable for pharmaceutical and biomedical processing. Hydrocortisone can suppress osteoblast function and thus reduce bone remodeling and impair bone tissue repair. The period of increased bone resorption is followed by reduction in bone formation. These occur by increasing apoptosis in osteoblasts through the production of reactive oxygen species (ROS), inhibition of bone formation transcription factors, and the accumulation of β-catenin leading to suppression of the Wnt/β-catenin pathway. CA13/HA50, CA13/HA/BER100, and CA/HA/ME100 composites nanofibers enhanced osteoblast functions proliferation, alkaline phosphates, calcium synthesis, bone formation transcription factors production that correlate with decreased β-catenin accumulation leading to the activation of the Wnt/β-catenin and Wnt/Ca(^2+^) pathways. The resorptive activity of osteoclasts cultured with different CA13 composites nanofibers was weaker than that of control osteoclastic cells. In addition, cell adhesion and proliferation were enhanced by the presence of CA13 composites fibers. Therefore, CA13/HA/BER100 and CA13/HA/ME100 may serve as valuable and novel scaffolds for osteoporotic bone regeneration.

## Figures and Tables

**Table 1:** Optimization of spinning conditions for different prepared nanofibers.

Sample Code	Cellulose Acetate Concentration (w%)	HA Concertation (mg)	HA/BER Composite Concertation (mg)	HA/ME Composite Concentration (mg)	Feeding Rate (mL/h)	Applied Voltage (KV)	Distance from Needle to Collector (cm)	Fiber Morphology
CA10	10	/	/	/	0.5	24	15	Beaded (Figure S1)
CA11	11	/	/	/	0.5	24	15	Beaded (Figure S1)
CA12	12	/	/	/	0.5	24	15	Beaded (Figure S1)
CA13	13	/	/	/	0.5	24	15	Unbeaded (Figure 1)
CA14	14	/	/	/	0.5	24	15	Beaded (Figure S1)
CA15	15	/	/	/	0.5	24	15	Unbeaded (Figure S1)
CA13/HA25	13	25	/	/	0.5	26	15	Unbeaded (Figure S2)
CA13/HA50	13	50	/	/	0.5	26	15	Unbeaded (Figure 1)
CA13/HA100	13	100	/	/	0.5	26	15	Beaded (Figure S2)
CA13/HA200	13	200	/	/	0.5	26	15	Precipitated (Figure S2)
CA13/HA/BER25	13	/	25	/	0.5	26	15	Unbeaded (Figure S3)
CA13/HA/BER50	13	/	50	/	0.5	27	15	Unbeaded (Figure S3)
CA13/HA/BER100	13	/	100	/	0.5	27	15	Unbeaded (Figure 1)
CA13/HA/BER200	13	/	200	/	0.5	27	15	Beaded (Figure S3)
CA13/HA/ME25	13	/	/	25	0.5	30	15	Unbeaded (Figure S4)
CA13/HA/ME50	13	/	/	50	0.5	30	15	Unbeaded (Figure S4)
CA13/HA/ME100	13	/	/	100	0.5	30	15	Unbeaded (Figure 1)
CA13/HA/ME200	13	/	/	200	0.5	30	15	Beaded (Figure S4)

**Table 2:** Primers’ sequence and qRT-PCR conditions.

Gene Name/Base Pair	Primer Sequence		Annealing Temperature (°C)	Number of Cycles
GAPDH	F	5′-AGATCCACAACGGATACATT-3′	52	35
	R	5′-TCCCTCAAGATTGTCAGCAA-3′		
DKK1	F	5′-GCTGCATGAGGCACGCTAT-3′	55	35
	R	5′-AGGGCATGCATATTCCGTTT-3′		
SOST	F	5′-GTGCAAGTGCAAGCGCCTCA-3′	60	40
	R	5′-GCTCCGCCTGGTTGGCTTTG-3′		
Sox9	F	5′-TCCAGCAAGAACAAGCCACA-3′	56	40
	R	5′-CGAAGGGTCTCTTCTCGCTC-3′		
RUNX2	F	5′-AGTGTGTGTGTCCGCATGAT-3′	56	40
	R	5′-CCACTTGGGGTCTAAGAACG-3′		
Osterix	F	5′-TGAGGAAGAAGCCCATTCAC-3′	53.5	40
	R	5′-ACTTCTTCTCCCGGGTGTG-3′		
COLA1	F	5′-CAAGGACTATGAAGTTGATGC-3′	43	40
	R	5′-ACCAGTAGAGAAATCGCAGT-3′		
OPG	F	5′-GTTCTTGCACAGCTTCACCA-3′	54	40
	R	5′-AAACAGCCCAGTGACCATTC-3′		
RANKL	F	5′-ACCAGCATCAAAATCCCAAG-3′	52	35
	R	5′-GGCCGCTAATTTCCTCACCA-3′		

**Table 3:** Shear viscosity, thickness, surface roughness, maximum strength, and maximum strain of the CA13 membranes before and after loading of nanocrystals.

Samples	Shear Viscosity (Pa·s)	Thickness (mm)	Surface Roughness Ra (µm)	Tensile Strength (MPa)	Maximum Strain (%)
CA13	2.399 ± 0.32	0.20 ± 0.04	0.11 ± 0.04	0.456 ± 0.23	2.160 ± 0.01
CA13/HA50	1.899 ± 0.34	0.24 ± 0.04	0.93 ± 0.51	1.016 ± 0.25	11.340 ± 0.02
CA13/HA/BER100	0.844 ± 0.50	0.22 ± 0.23	0.41 ± 0.23	1.278 ± 0.52	5.472 ± 0.01
CA13/HA/ME100	1.837 ± 0.35	0.21 ± 0.79	0.63 ± 0.79	1.302 ± 0.34	5.150 ± 0.01

The results are the means ± SD in each sample.

**Table 4:** Contact angle data, surface energy, porosity, and water uptake of the CA13 membranes before and after loading of nanocrystals.

Samples	Contact Angle (°)	Image	Surface Energy (mN/m)	Porosity (%)	Water Uptake (%)
CA13	113.46 ± 1.46	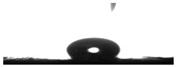	20.01 ± 0.86	0.89 ± 0.04	13.67 ± 0.65
CA13/HA50	53.46 ± 0.58	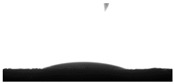	53.76 ± 0.96	0.98 ± 0.51	26.20 ± 0.46
CA13/HA/BER100	56.46 ± 0.56	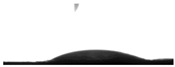	56.66 ± 0.92	0.99 ± 0.23	25.62 ± 0.56
CA13/HA/ME100	103.46 ± 1.04	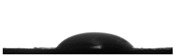	20.45 ± 0.86	1.01 ± 0.79	26.18 ± 0.58

The results are the means ± SD in each sample.

**Table 5:** Level of TRACP, calcium, total protein, and cell viability after 7 days of the treatment by CA13, CA13/HA, CA13/HA/BER and CA13/HA/ME fibers.

Groups		TRAcP (µg/mL)	Calcium Concentration (mg/mL)	Total Protein Concentration (mg/mL)	Cell Viability (%)
**Control osteoblast cells (OB)**		0.28 ± 0.06 a	0.46 ± 0.09 b	11.36 ± 0.90 c	100.10 ± 5.19 c
**OB**	CA13	0.33 ± 0.05 ab	0.92 ± 0.05 c	5.14 ± 0.73 a	86.60 ± 3.73 b
**OB**	CA13/HA50	0.41 ± 0.05 b	0.98 ± 0.07 c	16.02 ± 1.11 d	146.00 ± 2.69 d
**OB**	CA13/HA/BER100	0.39 ± 0.09 b	0.97 ± 0.05 c	9.79 ± 0.47 b	100.01 ± 1.65 c
**OB**	CA13/HA/ME100	0.45 ± 0.06 b	0.98 ± 0.07 c	11.93 ± 0.45 c	102.40 ± 2.79 c
**Induction osteoclast cells (OC)**		1.86 ± 0.04 g	0.21 ± 0.06 a	9.07 ± 0.76 b	75.03 ± 5.79 a
**OC**	CA13	1.23 ± 0.07 d	0.95 ± 0.04 c	5.21 ± 0.88 a	79.40 ± 2.59 a
**OC**	CA13/HA50	1.05 ± 0.09 c	0.90 ± 0.06 c	4.85 ± 1.49 a	76.77 ± 2.77 a
**OC**	CA13/HA/BER100	1.47 ± 0.05 f	0.94 ± 0.05 c	5.57 ± 0.70 a	86.85 ± 1.08 b
**OC**	CA13/HA/ME100	1.35 ± 0.06 e	0.96 ± 0.03 c	6.43 ± 0.84 a	83.75 ± 1.16 b

Results are the means of triplicate determination/each well ± SD in each group. Means with different small letters are significantly different at *p* < 0.05 for control OB groups and means with different capital letters are significantly different at *p* < 0.05 for the induction OC groups. Where: Control OB: Osteoblast cells, OB + CA 13%: Osteoblast cells treated with CA 13% fiber (cellulose acetate fiber), OB + CA13/HA50: Osteoblast cells treated with CA13/HA50 fiber (CA 13% fiber loaded with 50 mg n-HA), OB + CA13/HA/BER100: Osteoblast cells treated with CA13/HA/BER fiber (CA 13% fiber loaded with 100 mg n-HA/BER composite), and OB + CA13/HA/ME100: Osteoblast cells treated with CA13/HA/ME100 fiber (CA 13% fiber loaded with 100 mg n-HA/ME composite) all were incubated in differentiation media (DM) for 7 days. While induction OC: Osteoclast cells, OC + CA 13%: Osteoclast cells treated with CA 13% fiber (cellulose acetate fiber), OC + CA13/HA50: Osteoclast cells treated with CA13/HA50 fiber (CA 13% fiber loaded with 50 mg n-HA), OC + CA13/HA/BER100: Osteoclast cells treated with CA13/HA/BER100 fiber (CA 13% fiber loaded with 100 mg n-HA/BER composite), and OC+ CA13/HA/ME100: Osteoclast cells treated with CA13/HA/ME100 fiber (CA 13% fiber loaded with 100 mg n-HA/ME composite) all were incubated in differentiation media (DM) and 0.5 mg cortisol for 7 days.

**Table 6:** Effect of different treatments on Bone-MSCs markers of HCT-induced osteoclast formation.

Groups		Osteocalcin (ng/mL)	ALP Activity (%)	CD90 (ng/mg Protein)	P38-MAPK (ng/mg Protein)	PARP- ɣ (pg/mg Protein)
**Control osteoblast cells (OB)**		1.21 ± 0.03 e	21.82 ± 1.27 e	2.21 ± 0.03 c	0.43 ± 0.03 a	11.44 ± 0.3 a
**OB**	CA 13	1.53 ± 0.05 f	16.63 ± 0.82 d	2.01 ± 0.12 a	0.42 ± 0.02 a	12.23 ± 0.6 a
**OB**	CA13/HA50	2.14 ± 0.04 h	27.27 ± 1.21 g	2.23 ± 0.11 c	0.44 ± 0.03 a	15.31 ± 0.8 b
**OB**	CA13/HA/BER100	1.78 ± 0.07 g	21.82 ± 1.83 e	2.12 ± 0.12 b	0.40 ± 0.04 a	13.62 ± 0.8 ab
**OB**	CA13/HA/ME100	1.69 ± 0.07 g	24.54 ± 0.77 f	2.21 ± 0.03 c	0.57 ± 0.02 b	25.13 ± 0.6 c
**Induction osteoclast cells (OC)**		0.53 ± 0.08 a	05.45 ± 1.08 a	6.32 ± 0.28 g	2.53 ± 0.08 g	39.24 ± 0.6 f
**OC**	CA 13%	0.67 ± 0.05 b	06.36 ± 1.54 a	4.92 ± 0.19 f	2.43 ± 0.12 f	35.83 ± 0.7 f
**OC**	CA13/HA50	0.84 ± 0.07 c	10.91 ± 1.64 b	3.98 ± 0.25 e	1.73 ± 0.25 e	13.64 ± 0.7 ab
**OC**	CA13/HA/BER100	0.98 ± 0.07 d	13.63 ± 0.90 c	2.94 ± 0.17 d	1.45 ± 0.23 c	15.36 ± 0.6 b
**OC**	CA13/HA/ME100	0.86 ± 0.07 c	19.08 ± 1.55 e	3.08 ± 0.17 d	1.57 ± 0.22 d	13.73 ± 0.3 ab

Results are the means ± SD in each group. In each column, means with different letters are significantly different with each other where (a) the lowest mean, means with the same letters are significantly similar at *p* < 0.05.

**Table 7:** Effect of different treatments on osteoblast death markers of HCT-induced osteoclast formation.

Groups		Wnt-5 (ng/mg Protein)	GSK3βpS9 (µg/mg Protein)	GSK3β (µg/mg Protein)	GSK3βpS9/GSK3β	β.Catenin (pg/mg Protein)
**Control osteoblast cells (OB)**		2.48 ± 0.23 b	4.5 ± 0.23 a	6.8 ± 0.32 a	0.66	1.45 ± 0.005 c
**OB**	CA 13	2.58 ± 0.25 b	4.4 ± 0.21 a	6.3 ± 0.22 a	0.69	1.32 ± 0.003 a
**OB**	CA13/HA50	2.85 ± 0.26 c	4.5 ± 0.11 a	6.7 ± 0.23 a	0.67	1.42 ± 0.001 bc
**OB**	CA13/HA/BER100	2.73 ± 0.23 c	3.9 ± 0.23 a	6.8 ± 0.19 a	0.57	1.37 ± 0.007 b
**OB**	CA13/HA/ME100	2.17 ± 0.22 a	3.7 ± 0.21 a	6.1 ± 0.23 a	0.61	1.46 ± 0.003 c
**Induction osteoclast cells (OC)**		7.85 ± 0.32 g	16.2 ± 0.89 e	15.3 ± 0.89 e	1.06	3.97 ± 0.023 g
**OC**	CA 13%	5.93 ± 0.23 f	9.8 ± 1.50 d	13.0 ± 0.77 d	0.75	3.63 ± 0.015 f
**OC**	CA13/HA50	5.17 ± 0.25 e	9.0 ± 0.97 d	10.9 ± 0.72 c	0.82	2.87 ± 0.009 e
**OC**	CA13/HA/BER100	3.73 ± 0.29 d	7.5 ± 1.35 b	7.6 ± 0.93 b	0.99	1.98 ± 0.008 d
**OC**	CA13/HA/ME100	4.01 ± 0.10 e	8.8 ± 1.26 bc	10.0 ± 0.45 c	0.88	2.12 ± 0.003 d

Results are the means ± SD in each group. In each column, means with different letters are significantly different with each other, where (a) the lowest mean, means with the same letters are significantly similar at *p* < 0.05.

**Table 8:** Effect of different treatments on the expression of bone proliferation gene markers of HCT-induced osteoclast formation.

Groups		Osterix	Runx2	Cola1
**Control osteoblast cells (OB)**		1 ± 0.0000 e	1 ± 0.0000 e	1 ± 0.0000 e
**OB**	CA 13	1 ± 0.0020 e	1 ± 0.0015 e	1 ± 0.0023 e
**OB**	CA13/HA50	1.02 ± 0.11 e	1.02 ± 0.02 e	1.03 ± 0.03 e
**OB**	CA13/HA/BER100	1.12 ± 0.03 e	1.05 ± 0.01 e	1.09 ± 0.02 f
**OB**	CA13/HA/ME100	1.05 ± 0.01 e	1.04 ± 0.03 e	1.06 ± 0.02 e
**Induction osteoclast cells (OC)**		0.15 ± 0.03 a	0.27 ± 0.01 a	0.11 ± 0.01 a
**OC**	CA 13	0.23 ± 0.01 b	0.32 ± 0.03 b	0.13 ± 0.03 a
**OC**	CA13/HA50	0.63 ± 0.01 c	0.89 ± 0.02 c	0.59 ± 0.04 b
**OC**	CA13/HA/BER100	0.98 ± 0.01 d	0.99 ± 0.01 e	0.83 ± 0.03 d
**OC**	CA13/HA/ME100	0.97 ± 0.01 d	0.93 ± 0.03 d	0.78 ± 0.07 c

Results are the means ± SD in each group. In each column, means with different letters are significantly different with each other, where (a) the lowest mean, means with the same letters are significantly similar at *p* < 0.05.

**Table 9:** Effect of different treatments on bone resorption markers of HCT-induced osteoclast formation.

Groups		SOST	DKK	SOX9
**Control osteoblast cells (OB)**		1 ± 0.0000 a	1 ± 0.00000 a	1 ± 0.0000 a
**OB**	CA 13	0.98 ± 0.01 a	1.08 ± 0.022 b	1 ± 0.0016 a
**OB**	CA13/HA50	1.05 ± 0.02 b	1.06 ± 0.011 b	1.01 ± 0.03 a
**OB**	CA13/HA/BER100	1.12 ± 0.01 b	1.03 ± 0.032 b	1.07 ± 0.02 b
**OB**	CA13/HA/ME100	1.08 ± 0.03 b	1.08 ± 0.023 b	1.03 ± 0.01 a
**Induction osteoclast cells (OC)**		6.87 ± 0.03 g	12.25 ± 0.01 g	2.93 ± 0.05 f
**OC**	CA 13	5.12 ± 0.01 f	9.36 ± 0.031 f	2.81 ± 0.07 e
**OC**	CA13/HA50	4.23 ± 0.03 e	7.23 ± 0.032 e	2.15 ± 0.03 d
**OC**	CA13/HA/BER100	3.96 ± 0.07 d	5.87 ± 0.042 d	1.56 ± 0.07 c
**OC**	CA13/HA/ME100	2.29 ± 0.03 c	4.26 ± 0.013 c	1.63 ± 0.03 c

Results are the means ± SD in each group. In each column, means with different letters are significantly different with each other where (a) the lowest mean, means with the same letters are significantly similar at *p* < 0.05.

**Table 10:** Effect of different treatments on RANKL pathway markers of DEXA-induced osteoclast formation.

Groups		RANKL	OPG	RANKL/OPG
Control osteoblast cells (OB)		1 ± 0.0000 a	1 ± 0.0000 d	1 ± 0.0000 a
**OB**	CA 13	1.03 ± 0.02 a	0.99 ± 0.03 d	1.13 ± 0.01 b
**OB**	CA13/HA50	0.98 ± 0.01 a	0.98 ± 0.02 d	1.42 ± 0.04 d
**OB**	CA13/HA/BER100	1.08 ± 0.03 a	1.32 ± 0.01 e	1.22 ± 0.03 c
**OB**	CA13/HA/ME100	1.04 ± 0.02 a	1.23 ± 0.12 e	1.15 ± 0.01 b
Induction osteoclast cells (OC)		13.7 ± 0.92 f	0.41 ± 0.01 a	23.5 ± 1.32 g
**OC**	CA 13	11.1 ± 1.12 e	0.45 ± 0.03 a	21.2 ± 1.17 g
**OC**	CA13/HA50	8.32 ± 0.47 d	0.74 ± 0.04 b	14.3 ± 1.02 f
**OC**	CA13/HA/BER100	3.56 ± 0.08 b	0.97 ± 0.03 d	9.38 ± 0.08 e
**OC**	CA13/HA/ME100	4.57 ± 0.09 c	0.83 ± 0.02 c	10.2 ± 0.03 e

Results are the means ± SD in each group. In each column, means with different letters are significantly different with each other where (a) the lowest mean, means with the same letters are significantly similar at *p* < 0.05.

## Data Availability

The data presented in this study are contained within the article and the supplementary material.

## Supplementary Materials

The following are available online at https://www.mdpi.com/article/10.3390/polym13234140/s1, Figure S1: SEM images of CA10, CA11, CA12, CA14, and CA15 under 1000× and 3000× magnifications. Figure S2: SEM images of CA13/HA6.25, CA13/HA12.5, CA13/HA25, CA13/HA100, and CA13/HA200 under 1000× and 3000× magnifications. Figure S3: SEM images of CA13/HA/BER6.25, CA13/HA/BER12.5, CA13/HA/BER25, CA13/HA/BER50, and CA13/HA/BER200 under 1000× and 3000× magnifications. Figure S4: SEM images of CA13/HA/ME6.25, CA13/HA/ME12.5, CA13/HA/ME25, CA13/HA/ME50, and CA13/HA/ME200 under 1000× and 3000× magnifications.
