# Supplementary material for: Cellulose Acetate Nanofibers: Incorporating Hydroxyapatite (HA), HA/Berberine or HA/Moghat Composites, as Scaffolds to Enhance In Vitro Osteoporotic Bone Regeneration
Source: Polymers (Basel). 2021 Nov 27;13(23):4140. doi: 10.3390/polym13234140 (PMC8659966; doi:10.3390/polym13234140)
Supplement: Supplementary file 1 [file polymers-13-04140-s001.zip › polymers-1442854-supplementary.pdf]

polymers

MDPI

Supplementary Materials

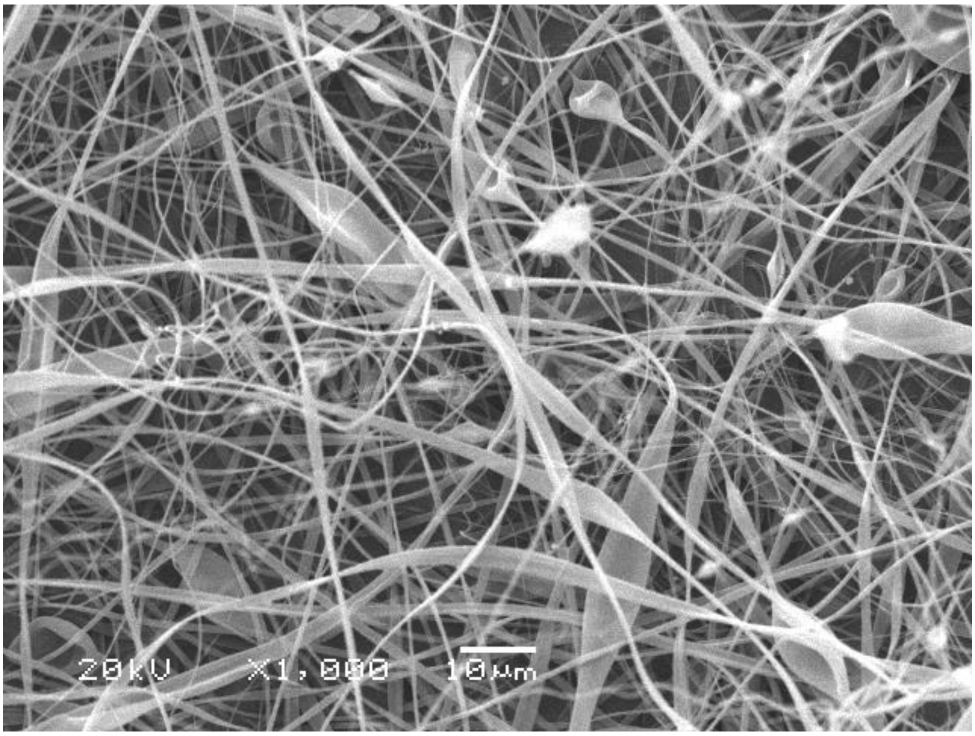

(**a**) CA10% 1000x

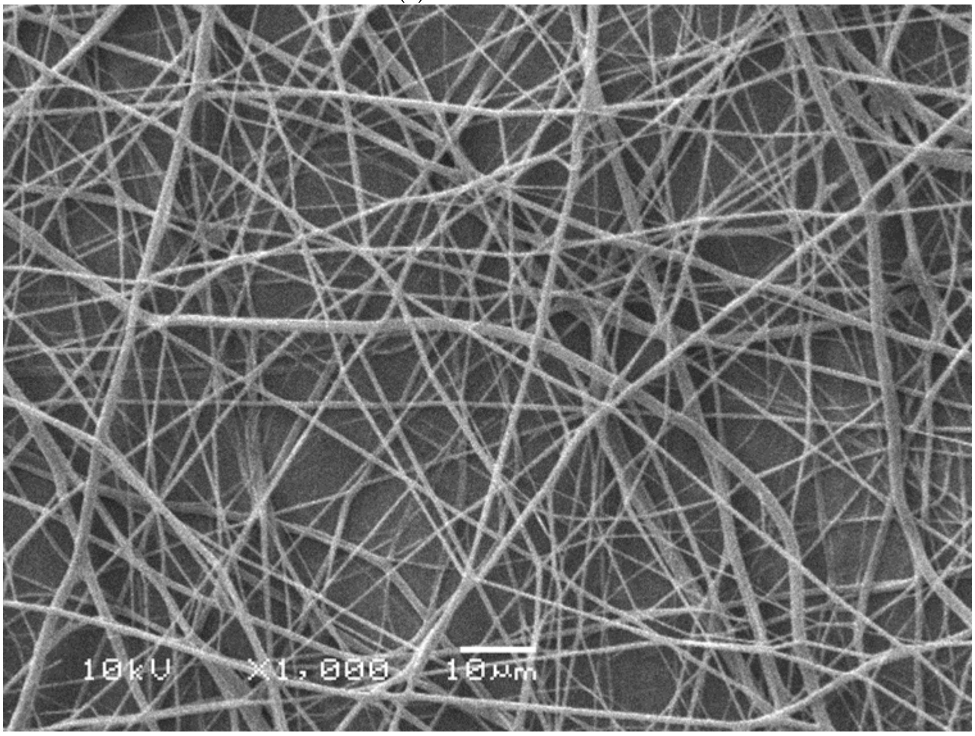

(**b**) CA11% 1000x

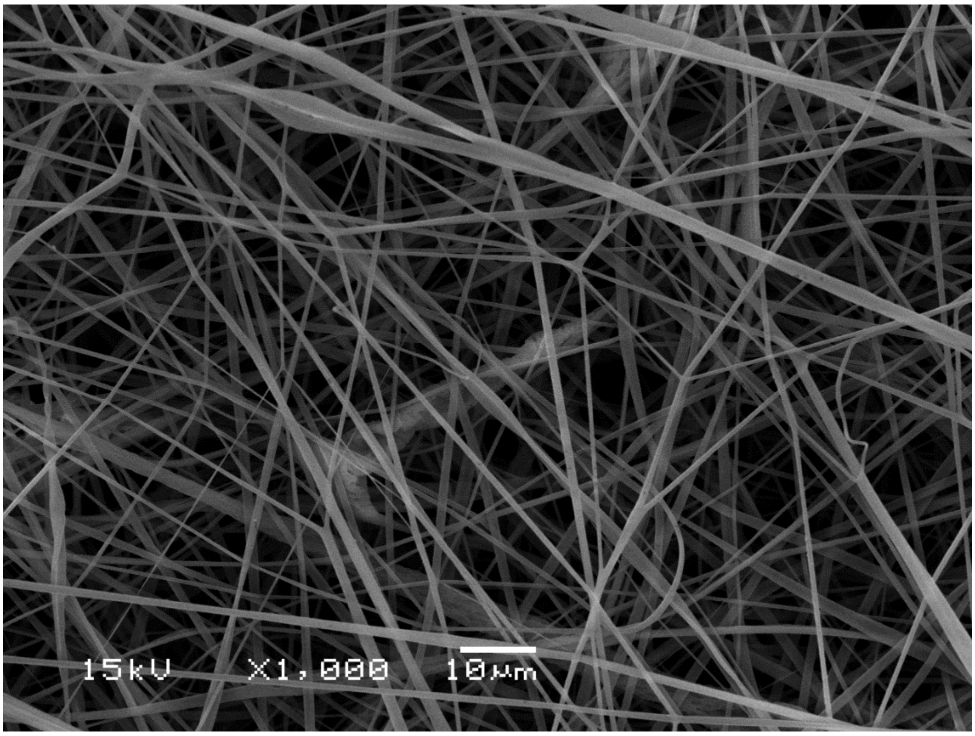

(**c**) CA12% 1000x

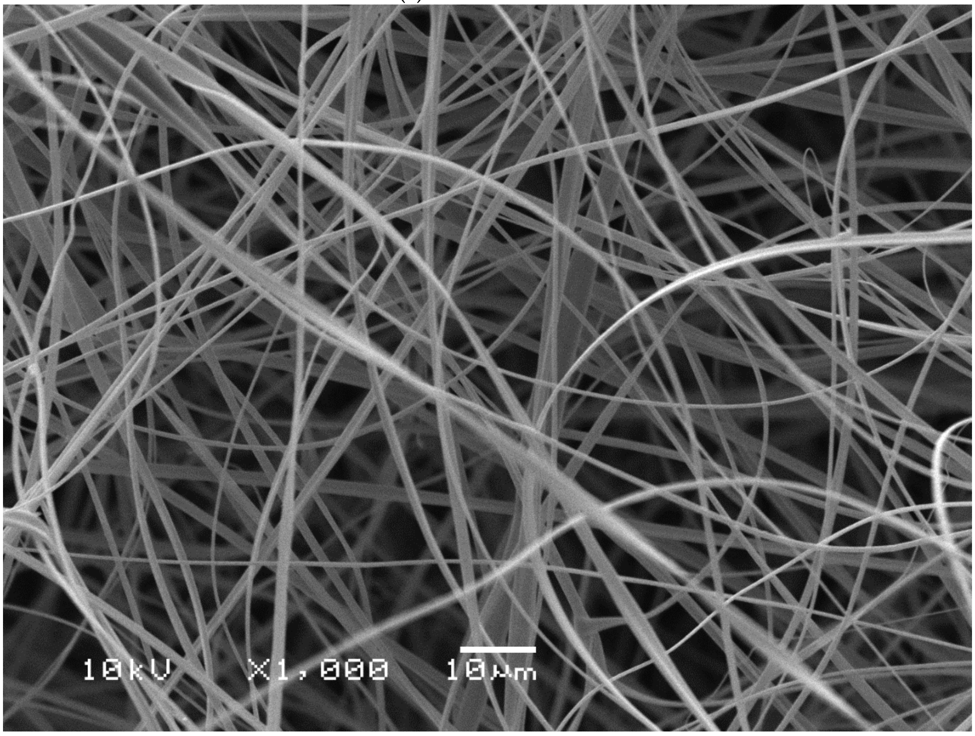

(**d**) CA14% 1000x

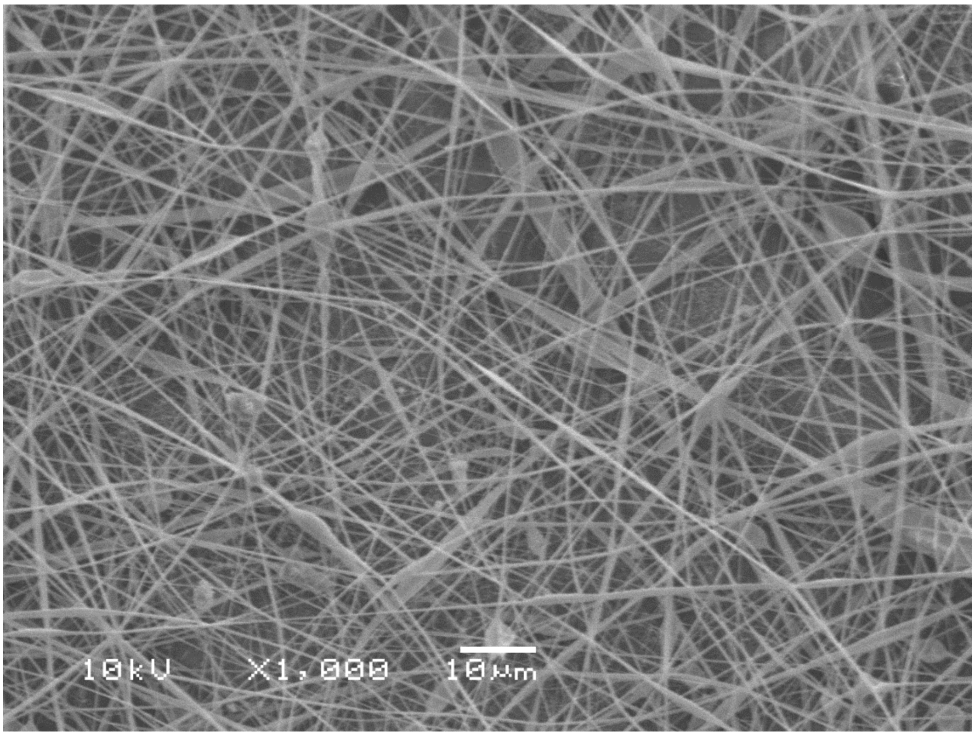

(**e**) CA15% 1000x

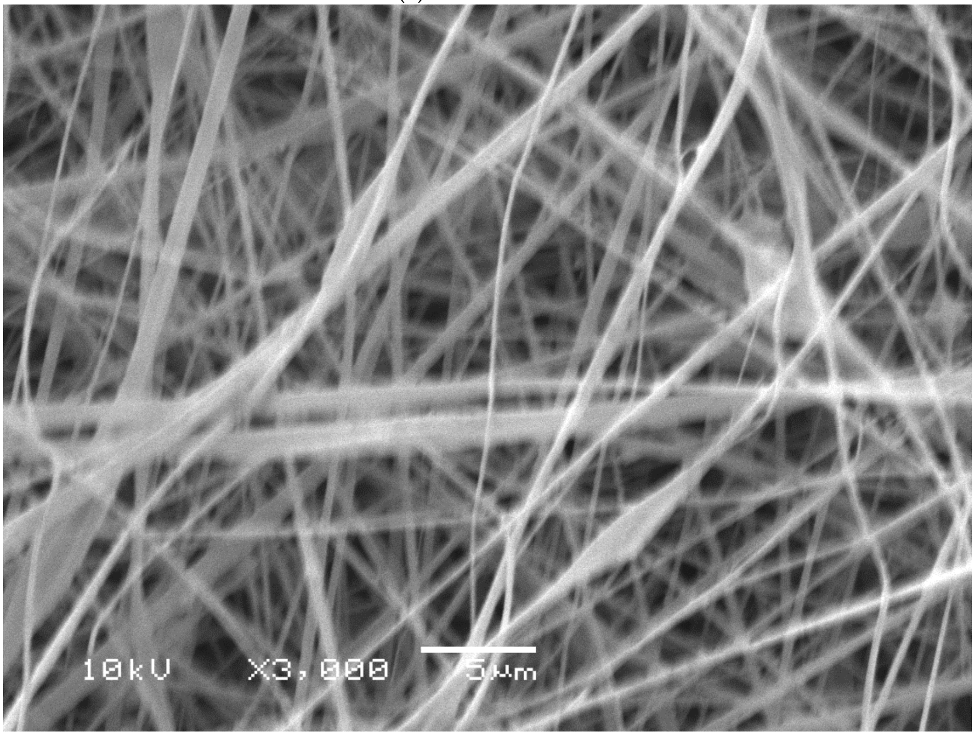

(**f**) CA10% 3000x

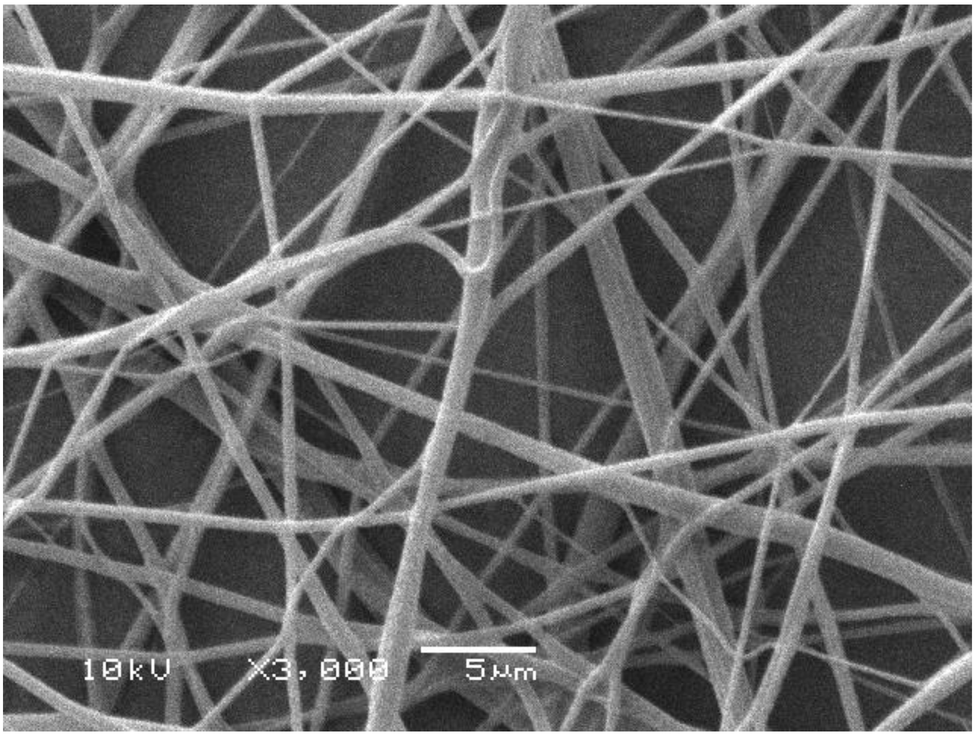

(g) CA11% 3000x

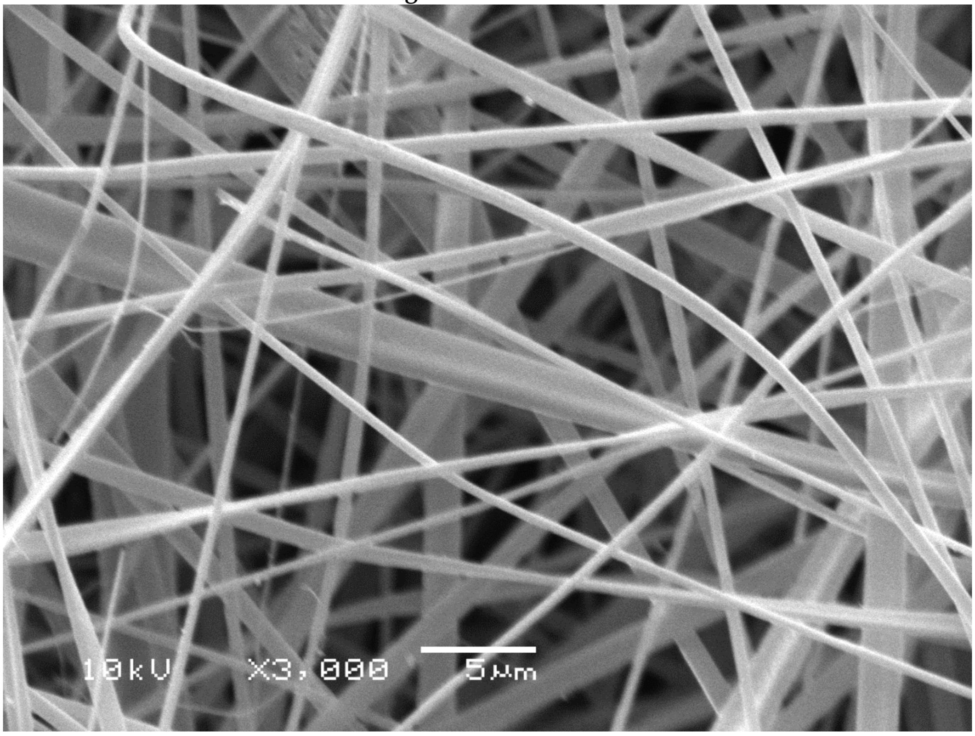

(h) CA12% 3000x

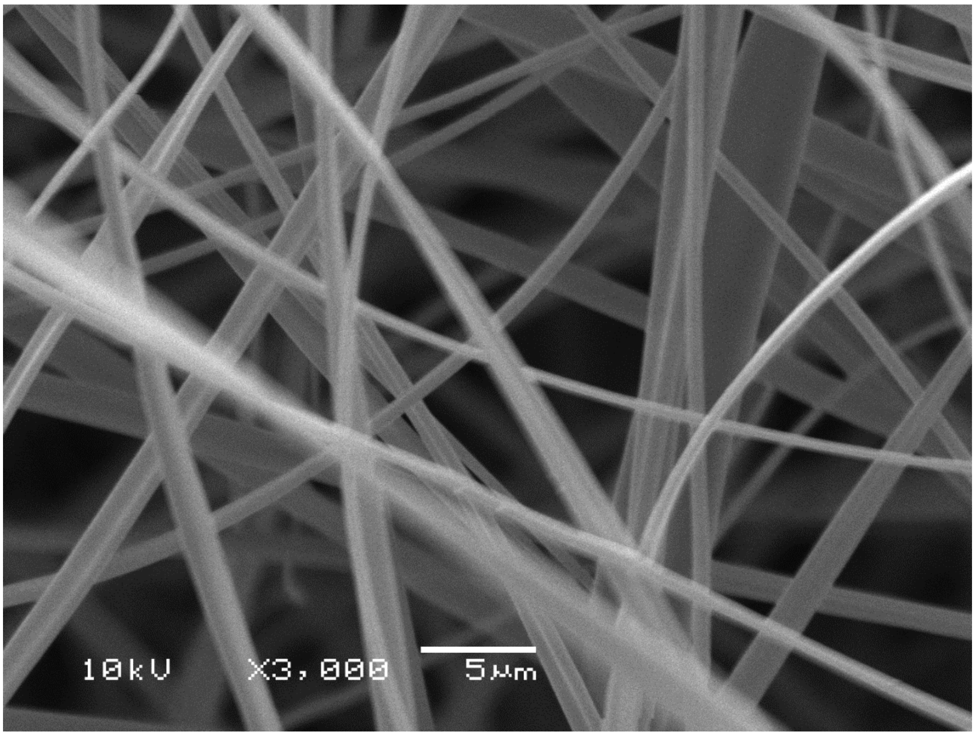

(i) CA14% 3000x

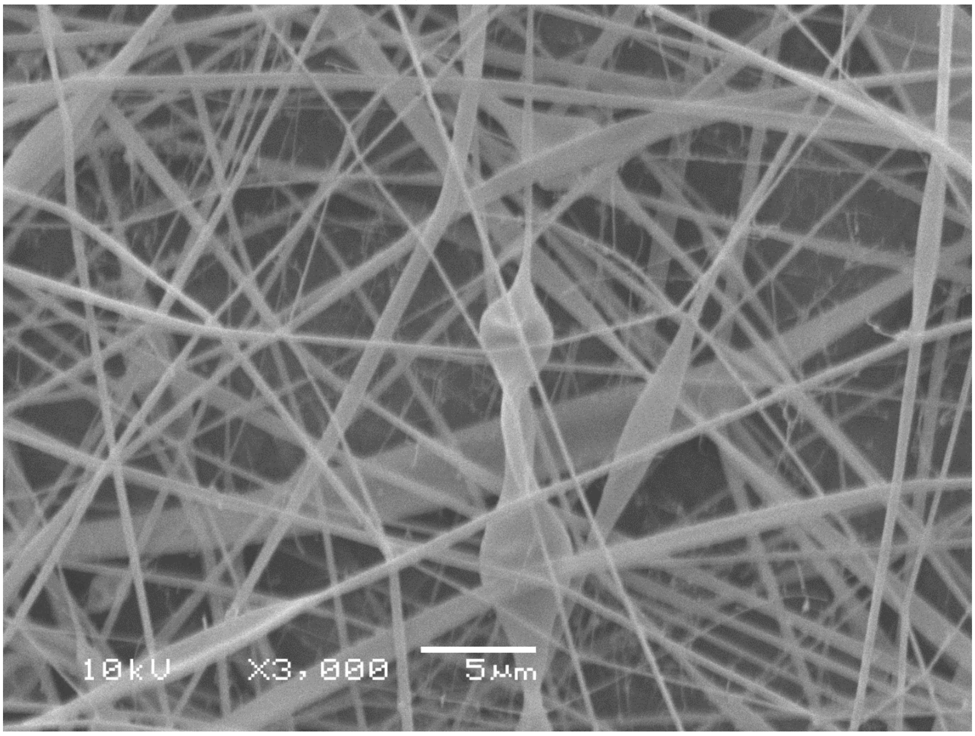

(j) CA15% 3000x

Figure S1. SEM images of CA10, CA11, CA12, CA14, and CA15 under 1000x (**a**–**e**) and 3000x (**f**–**j**) magnifications.

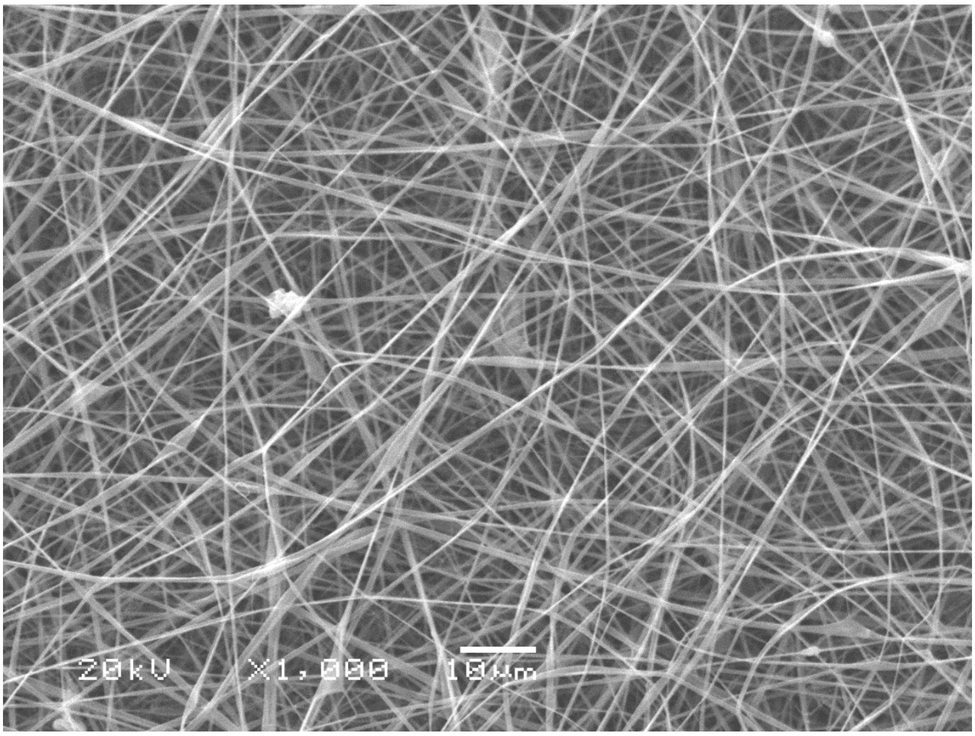

(**a**) CA13%-HA 6.25 mg 1000x

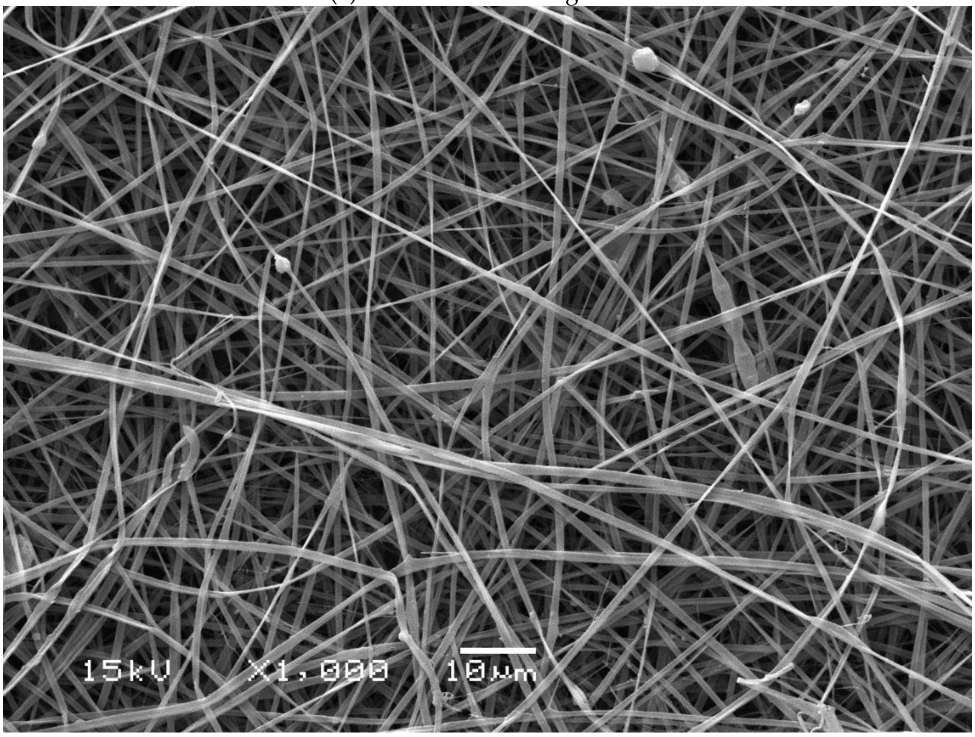

(**b**) CA13%-HA 12.5 mg 1000x

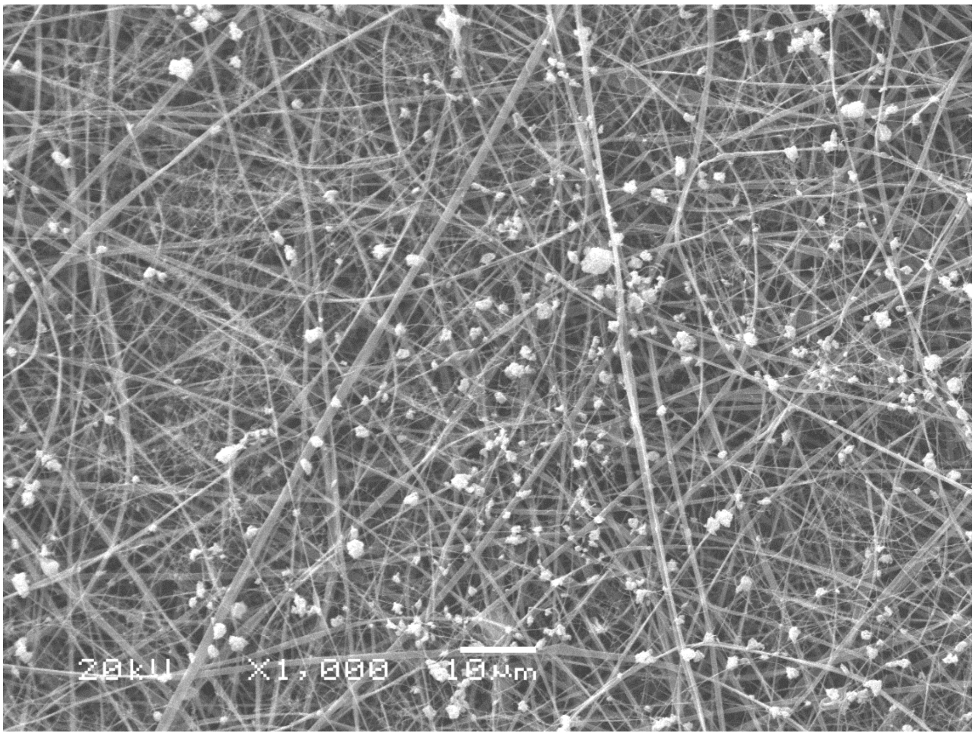

(**c**) CA13%-HA 25 mg 1000x

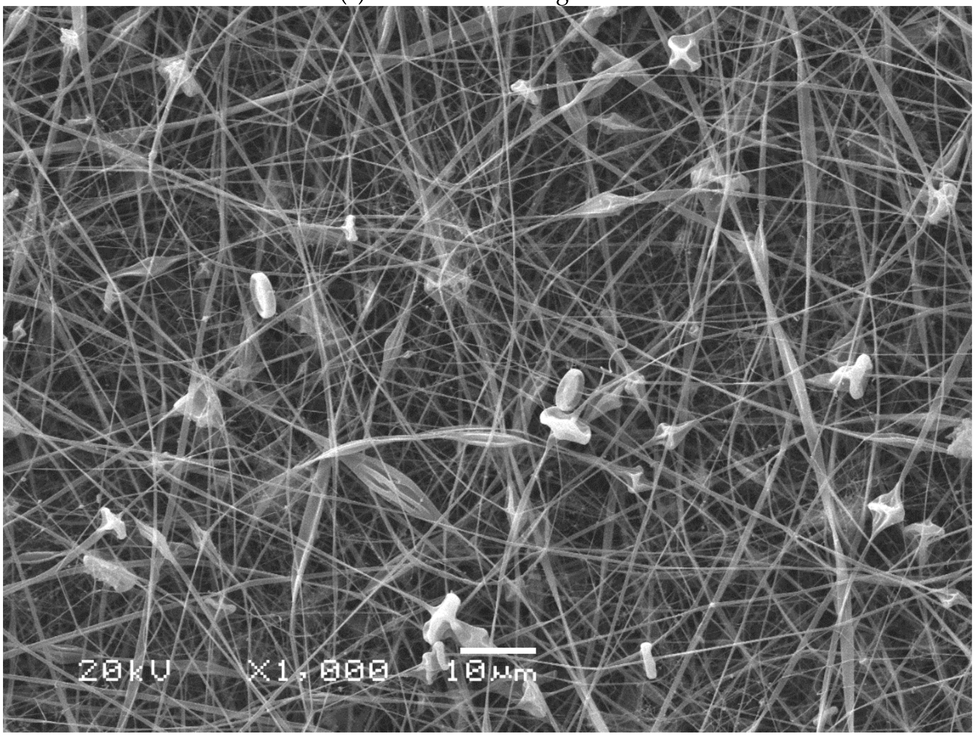

(**d**) CA13%-HA 100 mg 1000x

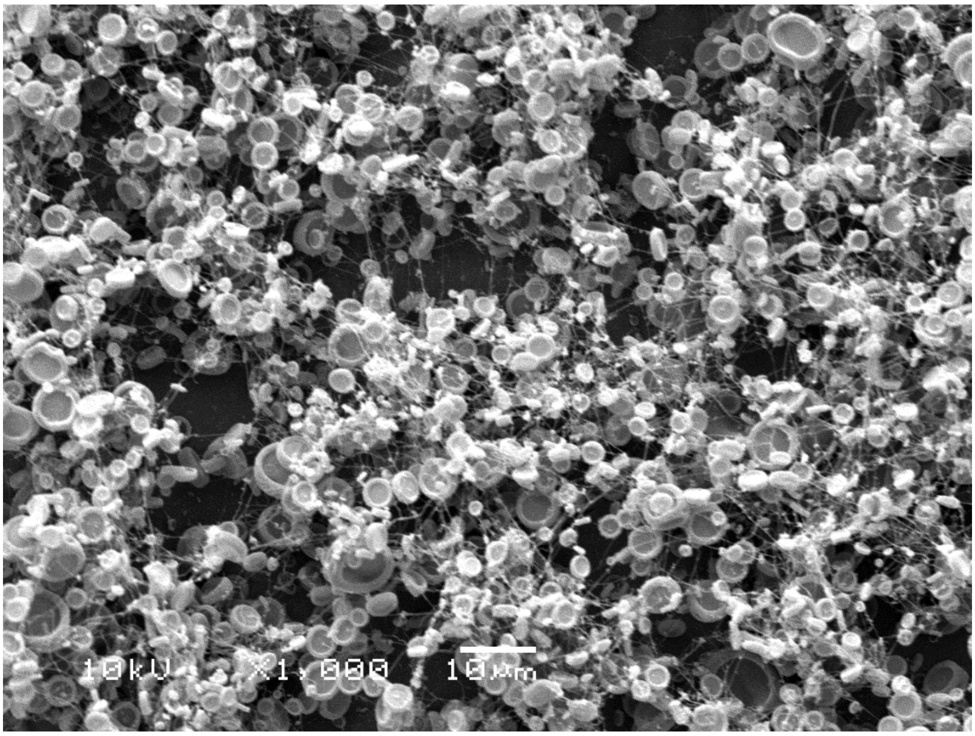

(**e**) CA13%-HA 200 mg 1000x

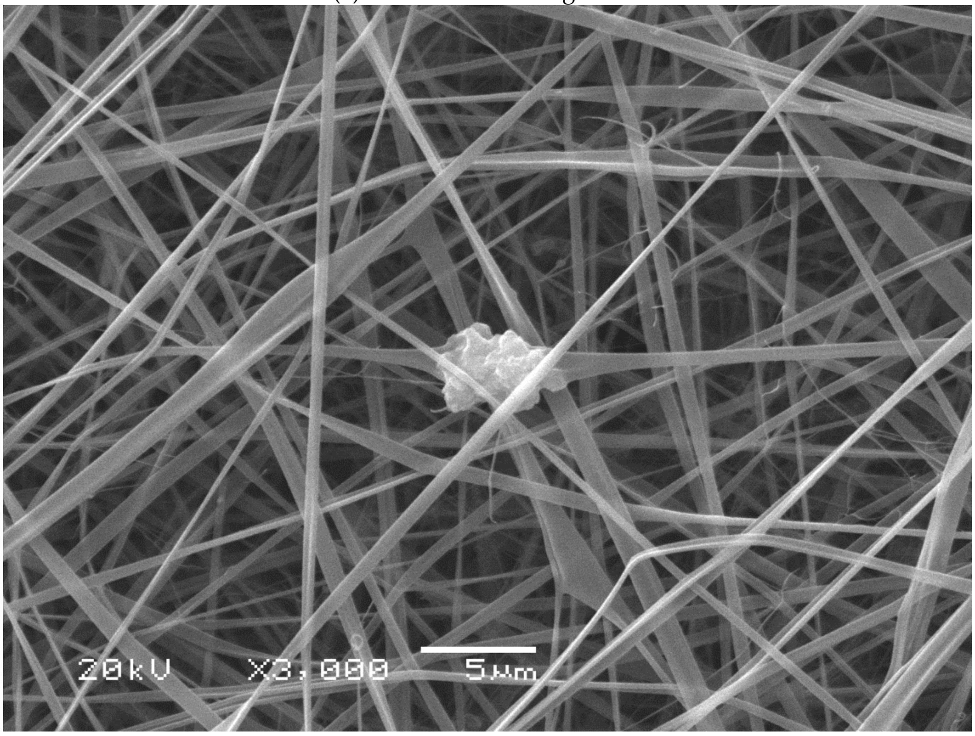

(**f**) CA13%-HA 6.25 mg 3000x

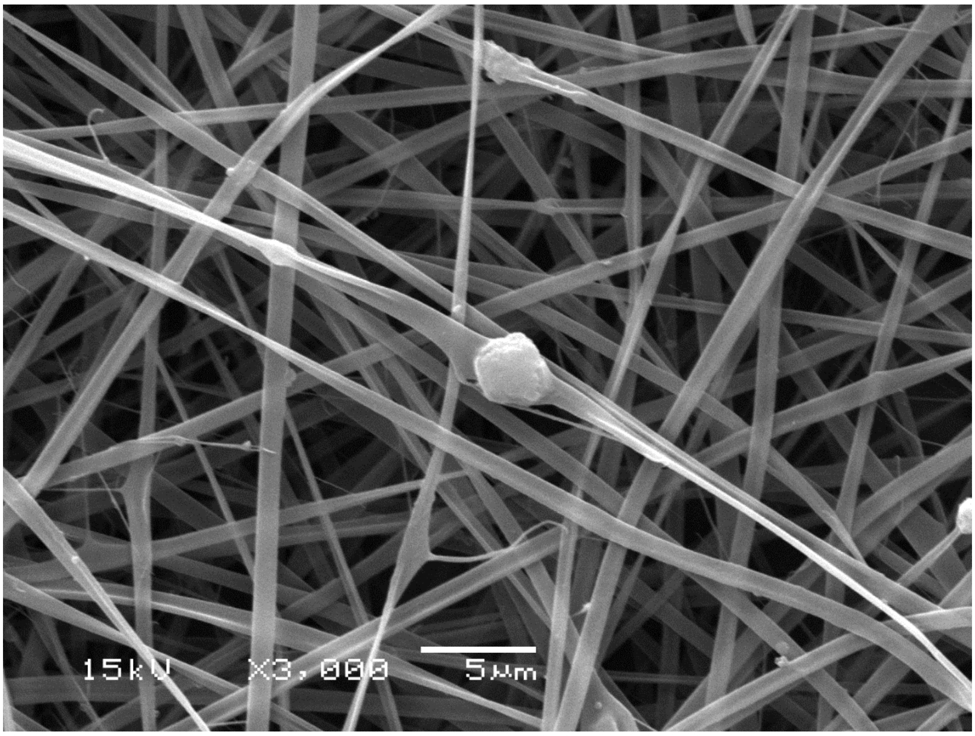

(**g**) CA13%-HA 12.5 mg 3000x

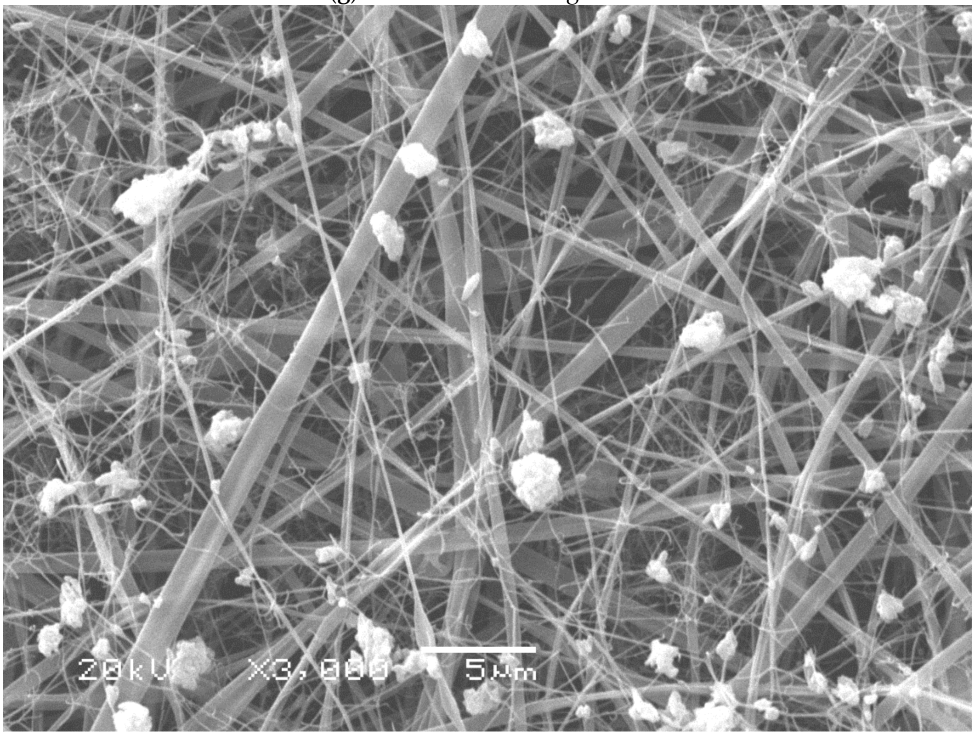

(**h**) CA13%-HA 25 mg 3000x

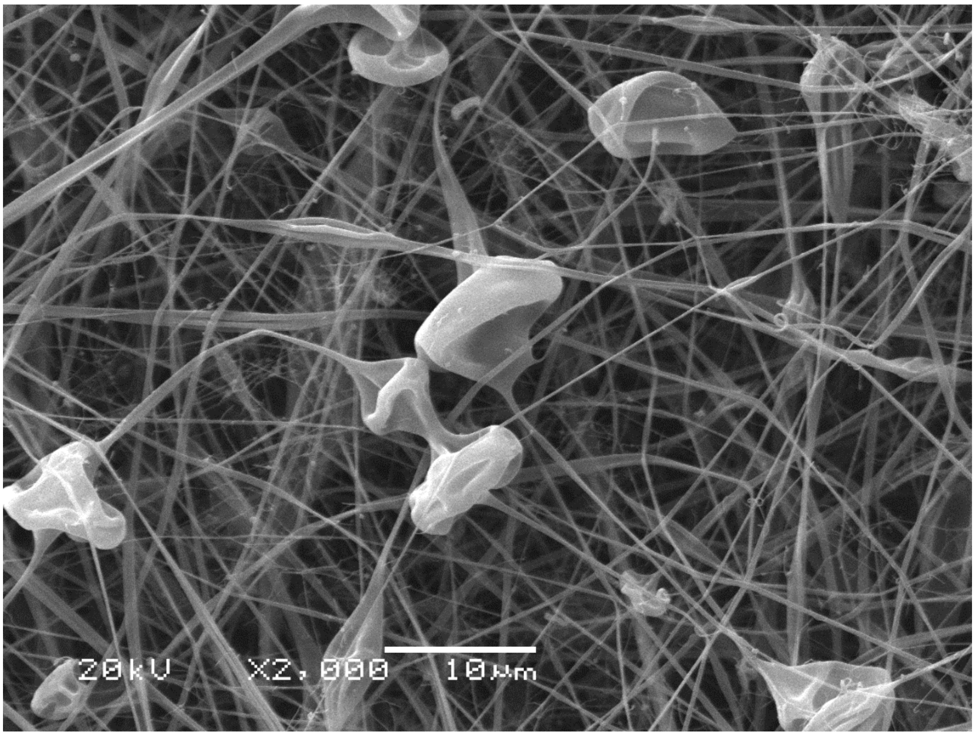

(**i**) CA13%-HA 100 mg 2000x

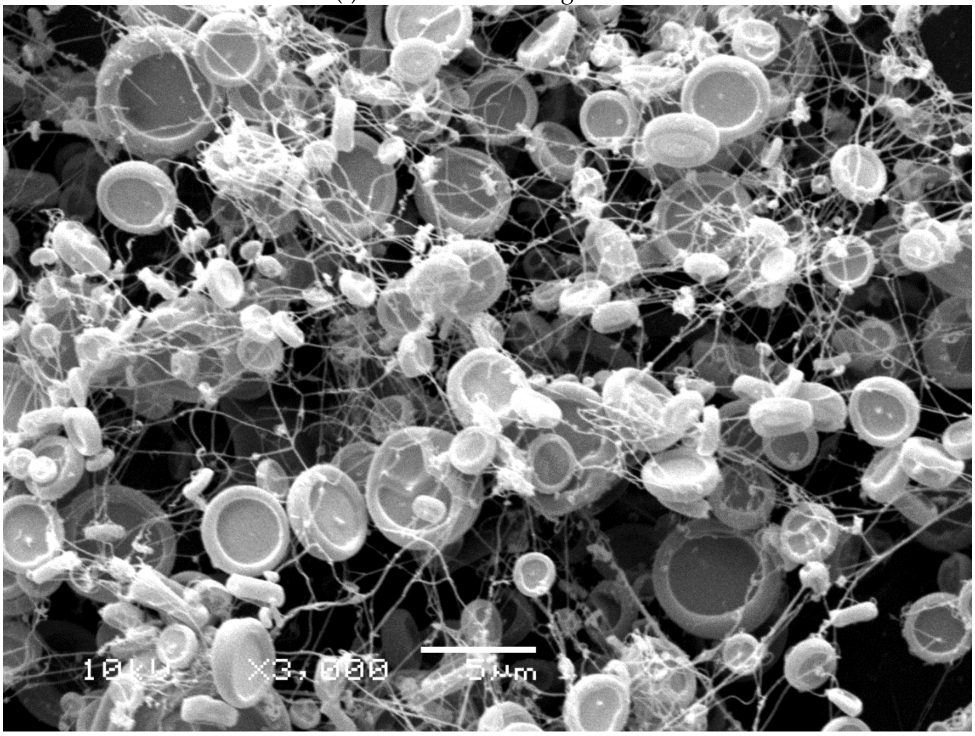

(**j**) CA13%-HA 200 mg 3000x

Figure S2. SEM images of CA13/HA6.25mg, CA13/HA12.5mg, CA13/HA25mg, CA13/HA100mg, and CA13/HA200mg under 1000× (**a**–**e**) and 3000× (**f**–**j**) magnifications.

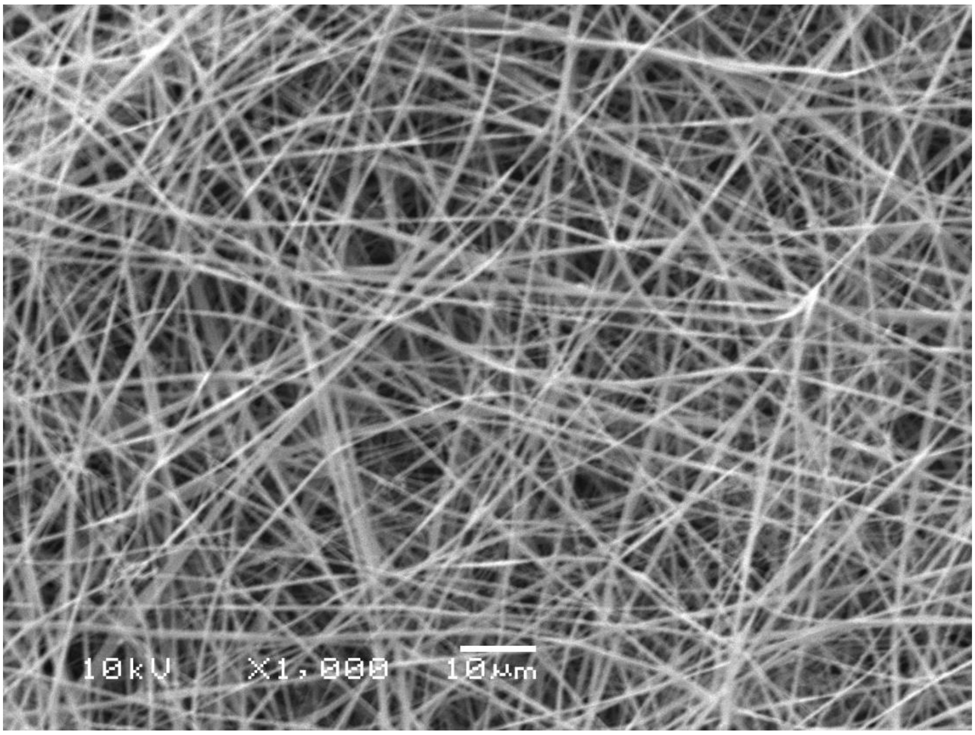

(**a**) CA13%-HA+BER 6.25 mg 1000x

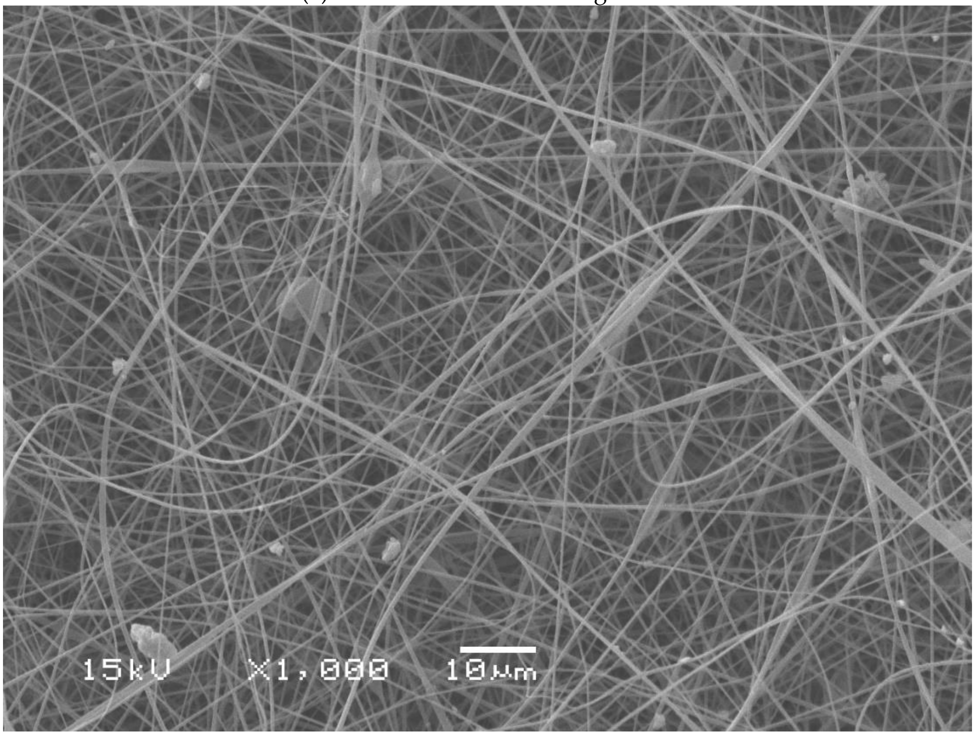

(**b**) CA13%-HA+BER 12.5 mg 1000x

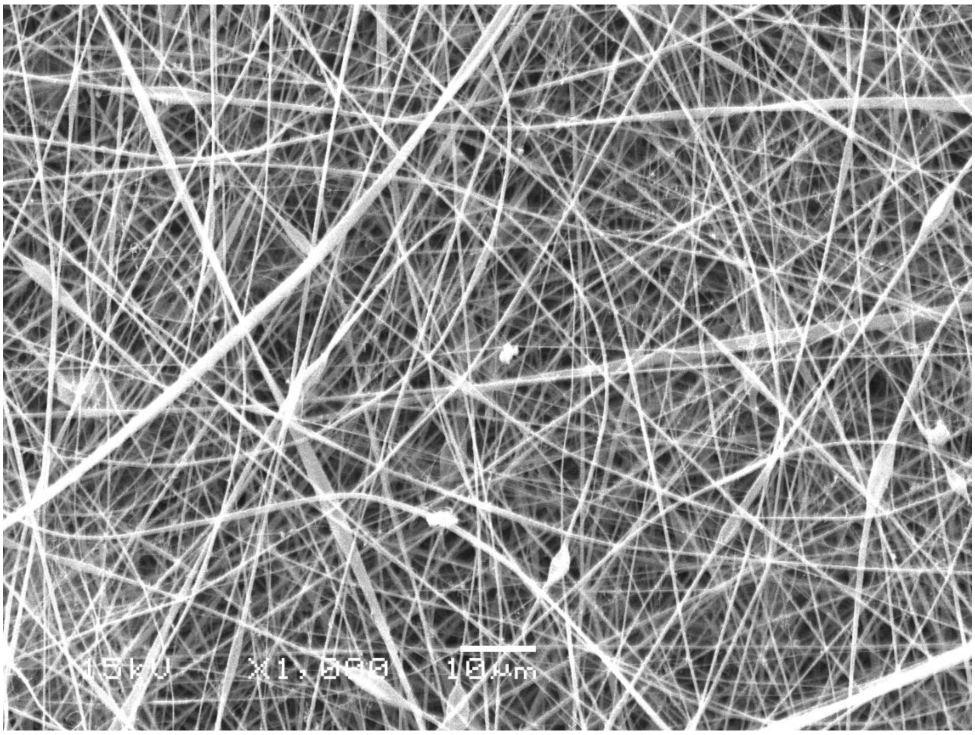

(**c**) CA13%-HA+BER 25 mg 1000x

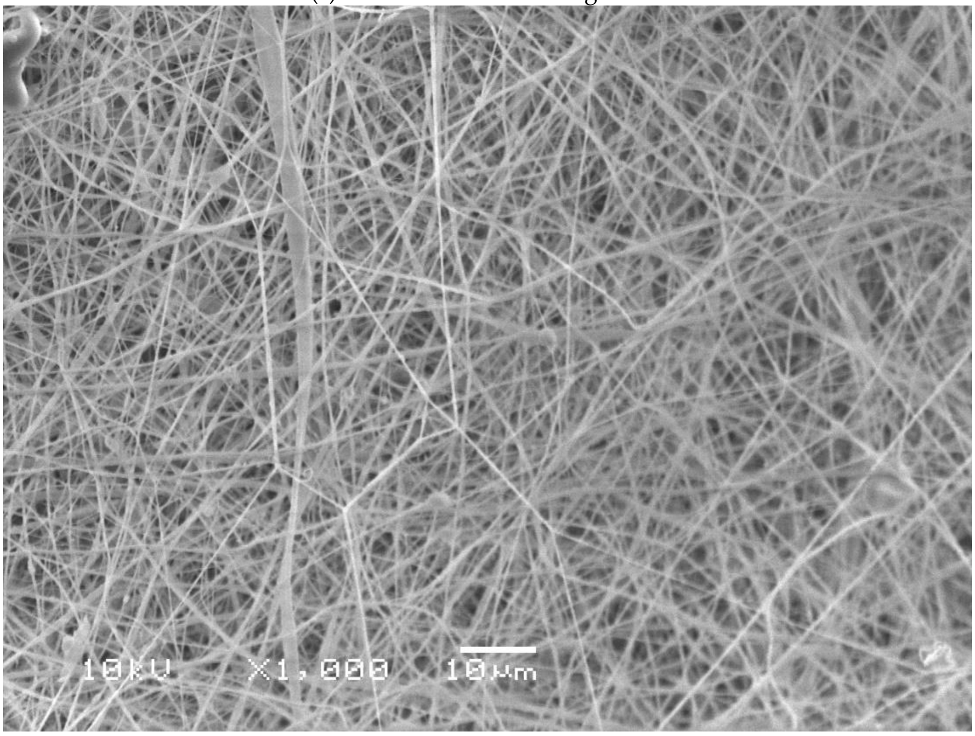

(**d**) CA13%-HA+BER 50 mg 1000x

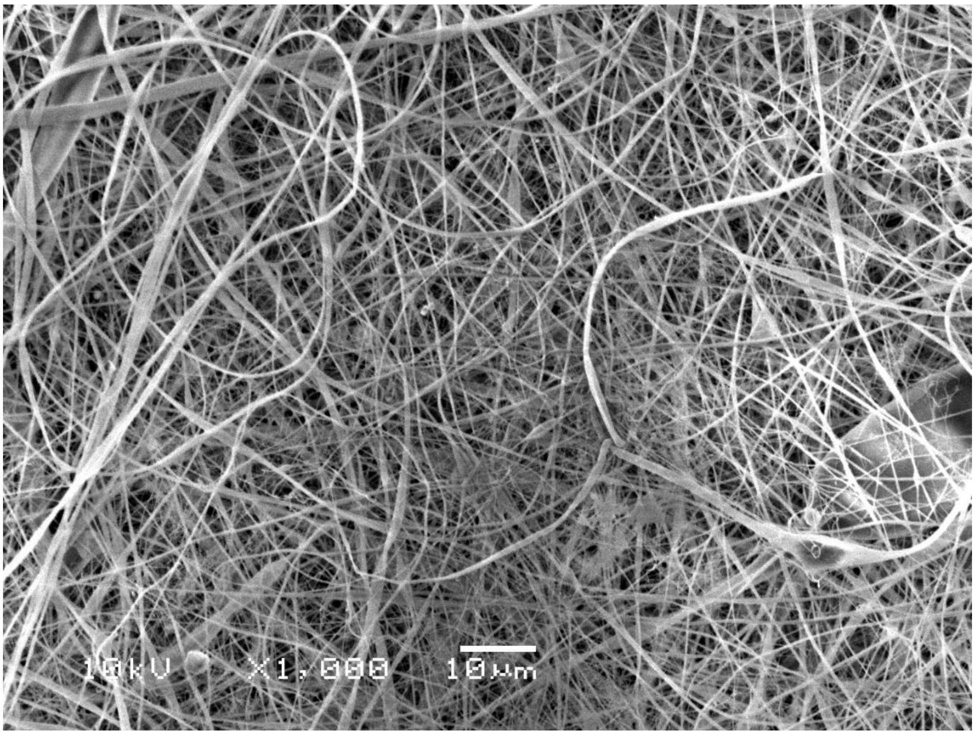

(**e**) CA13%-HA+BER 200 mg 1000x 2

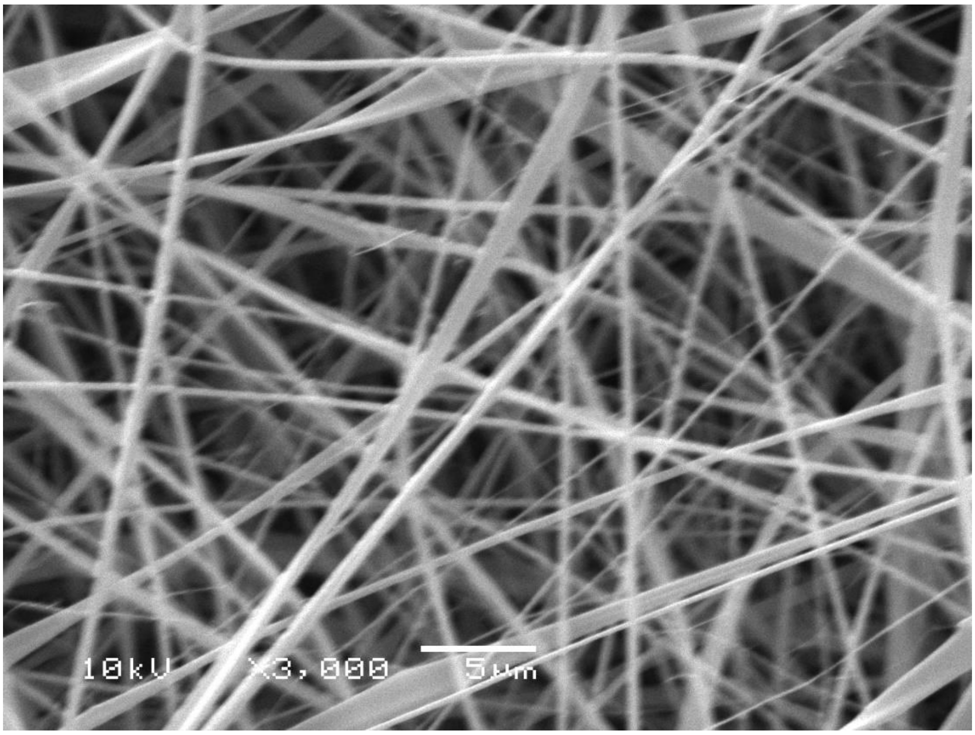

(**f**) CA13%-HA+BER 6.25 mg 3000x

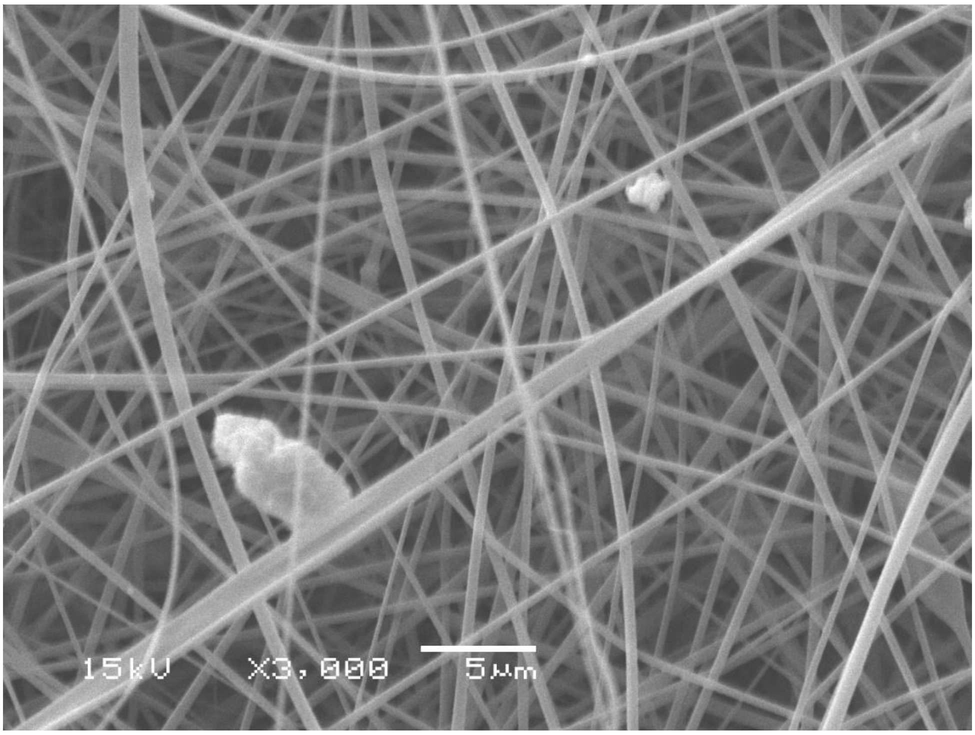

(**g**) CA13%-HA+BER 12.5 mg 3000x

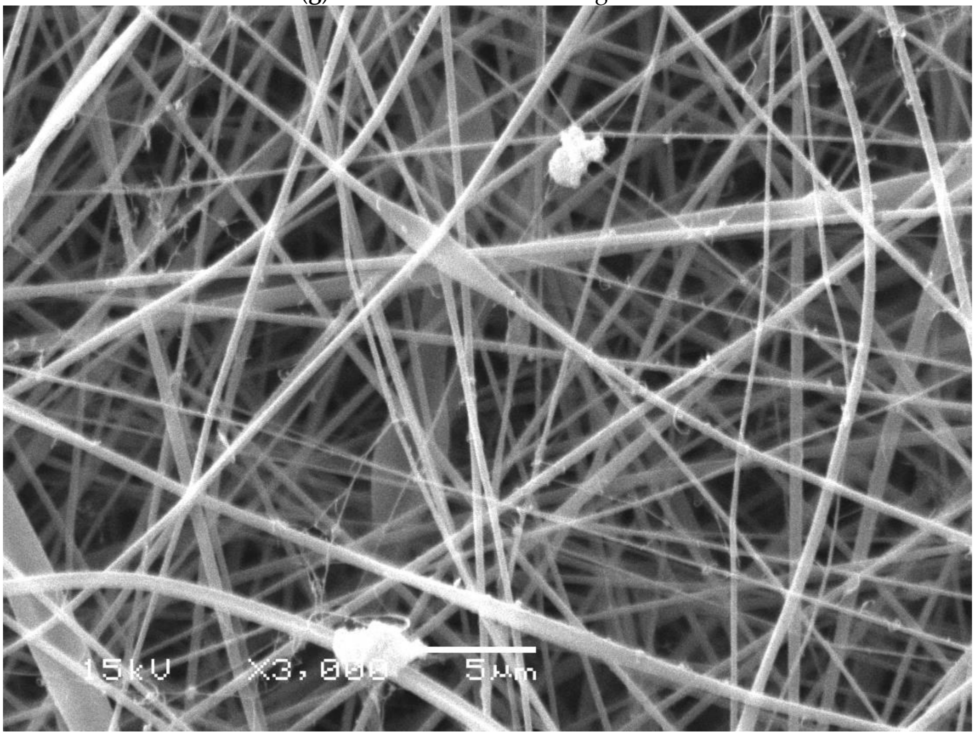

(**h**) CA13%-HA+BER 25 mg 3000x

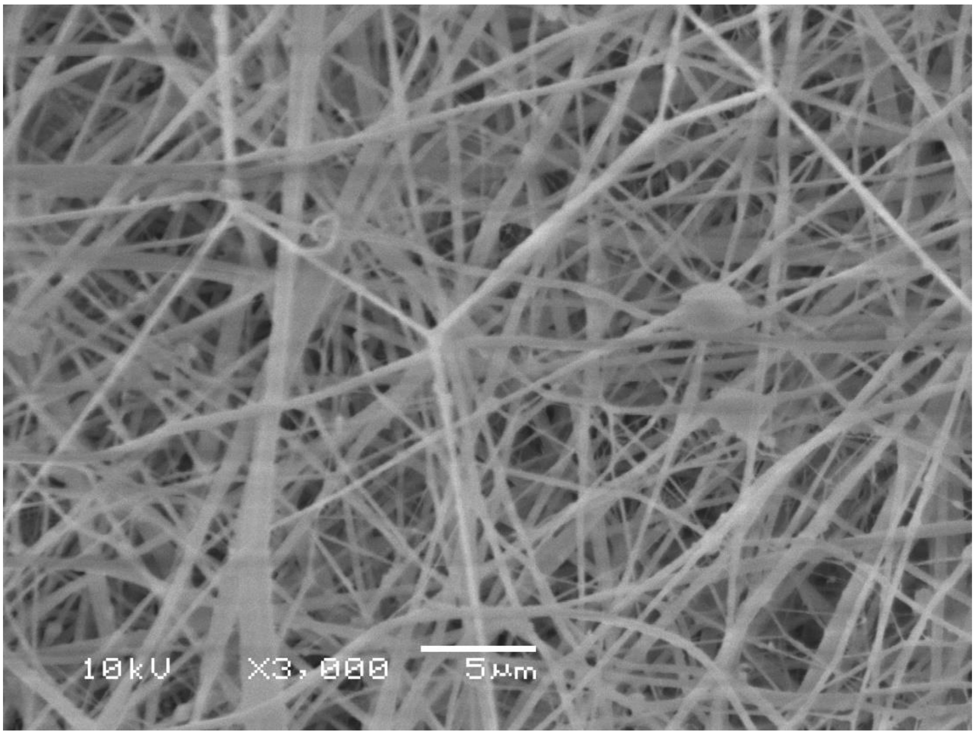

(**i**) CA13%-HA+BER 50 mg 3000x

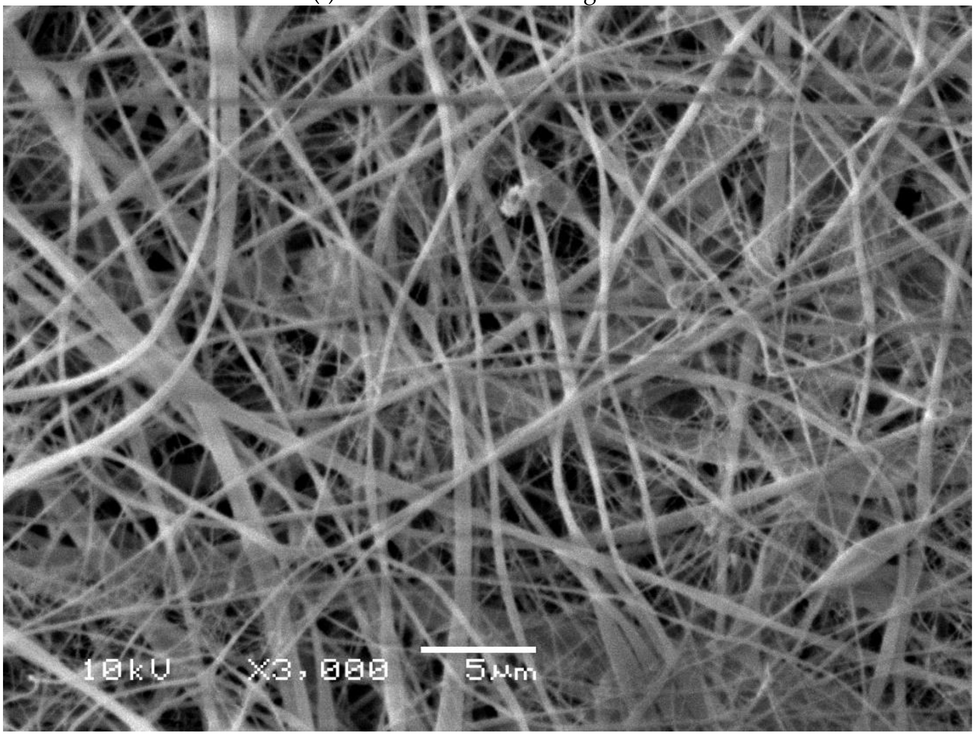

(**j**) CA13%-HA+BER 200 mg 3000x

Figure S3. SEM images of CA13/HA/BER6.25mg, CA13/HA/BER12.5mg, CA13/HA/BER25mg, CA13/HA/BER50mg, and CA13/HA/BER200mg under 1000× (**a**–**e**) and 3000× (**f**–**j**) magnifications.

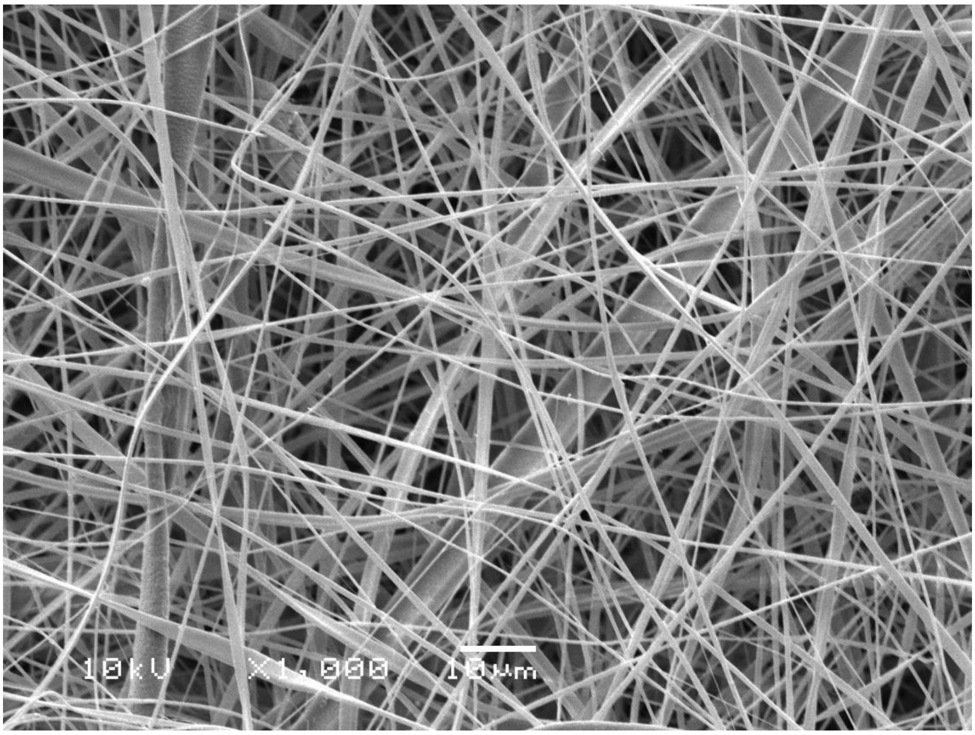

(**a**) CA13%-HA+ME 6.25 mg 1000x

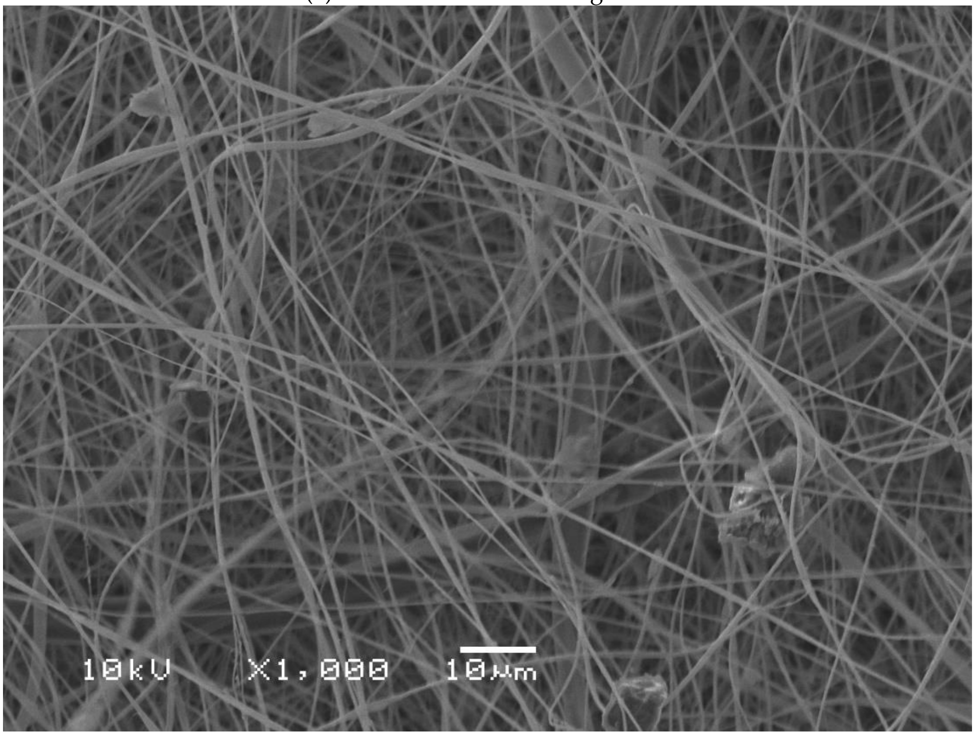

(**b**) CA13%-HA+ME 12.5 mg 1000x

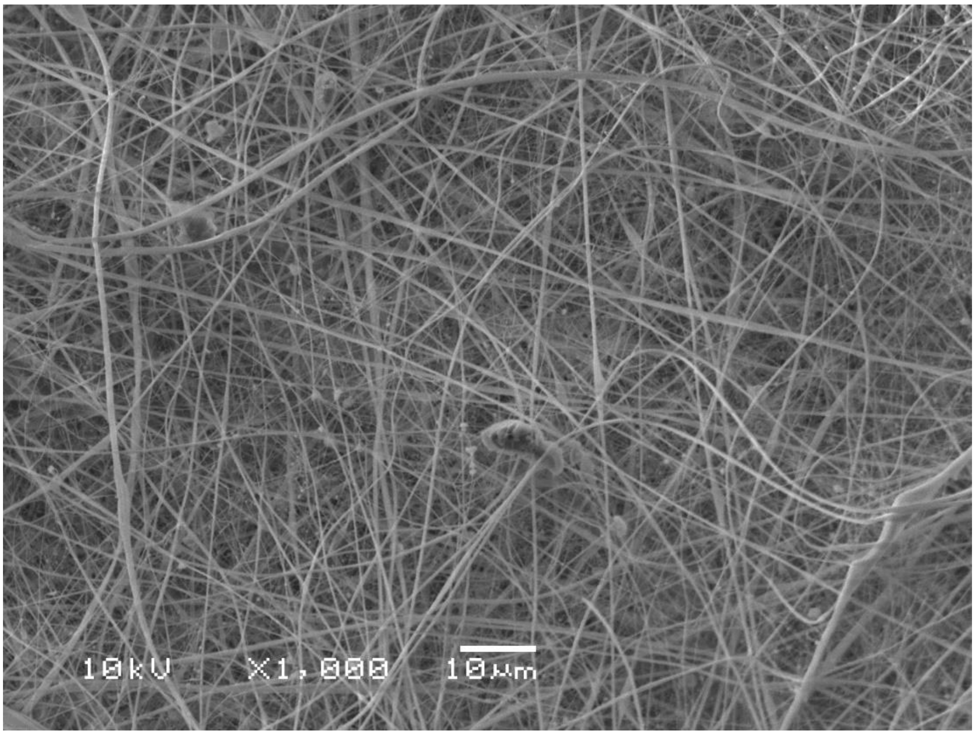

(**c**) CA13%-HA+ME 25 mg 1000x

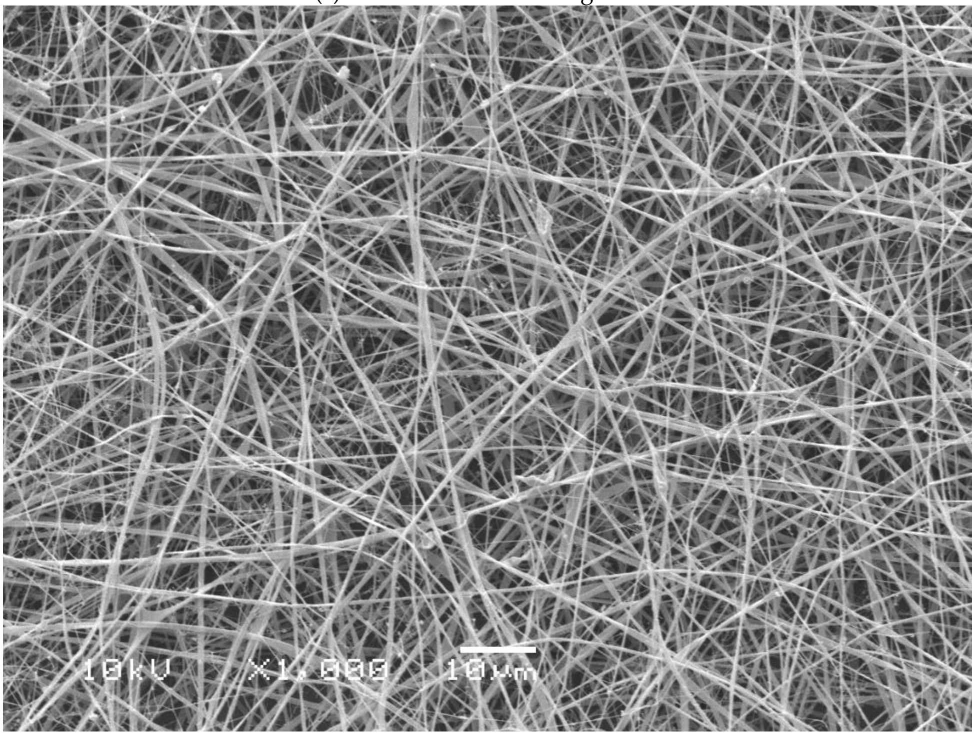

(**d**) CA13%-HA+ME 50 mg 1000x

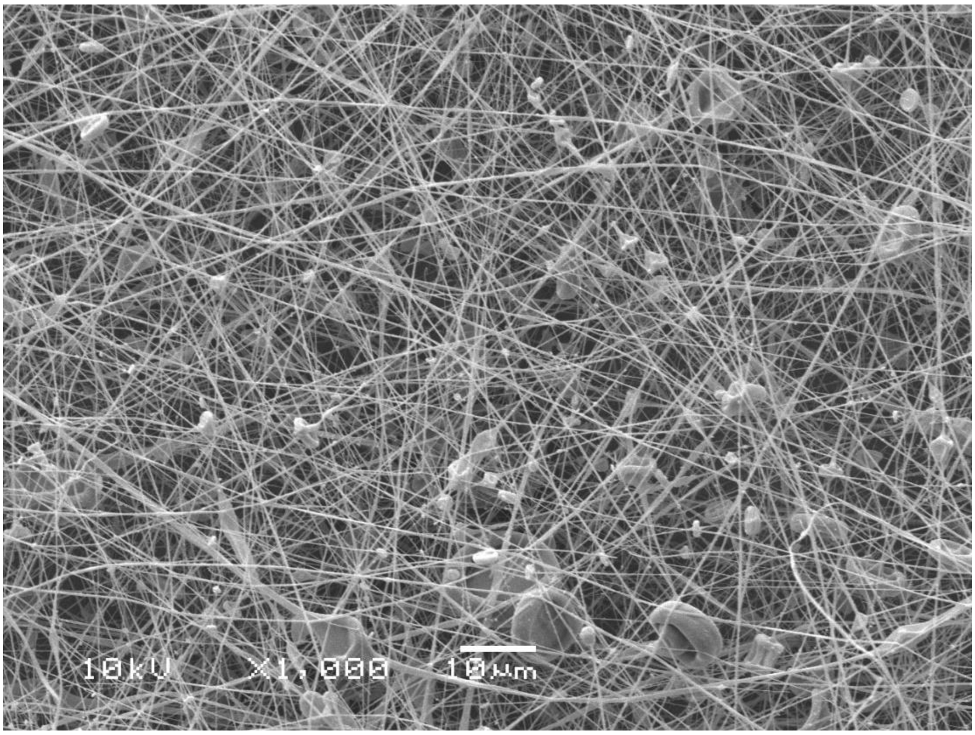

(**e**) CA13%-HA+ME 200 mg 1000x

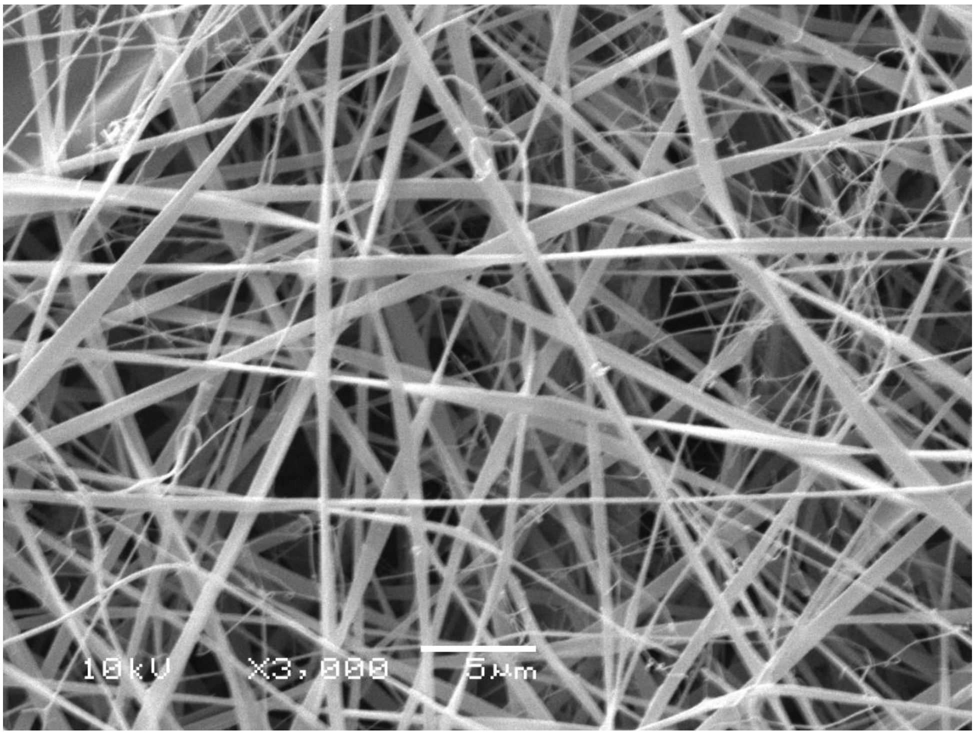

(**f**) CA13%-HA+ME 6.25 mg 3000x

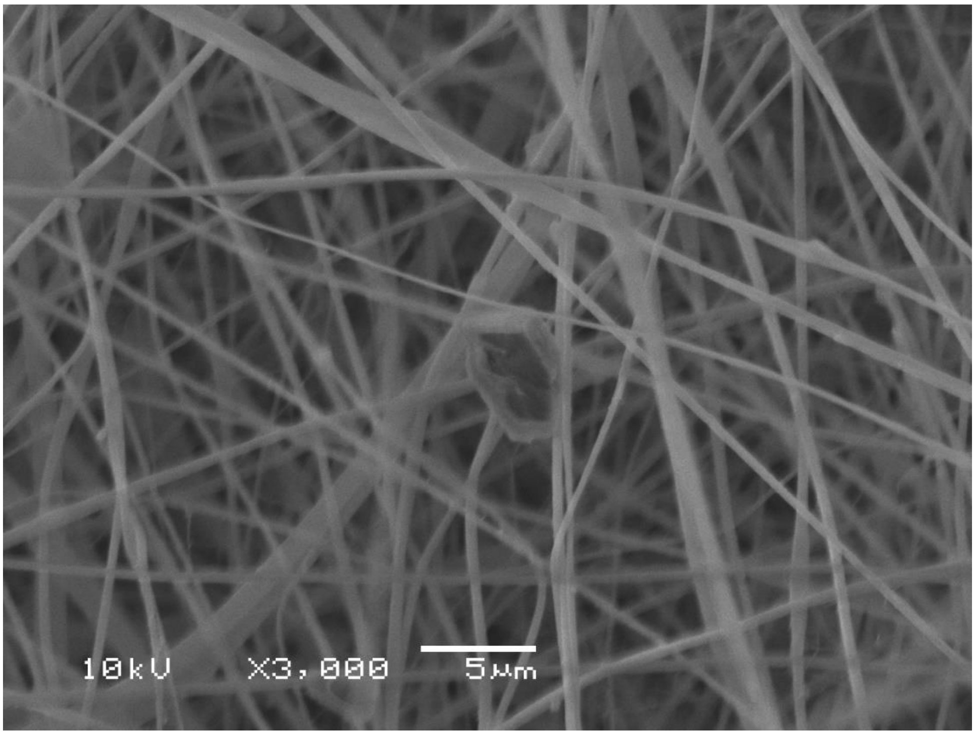

(**g**) CA13%-HA+ME 12.5 mg 3000x

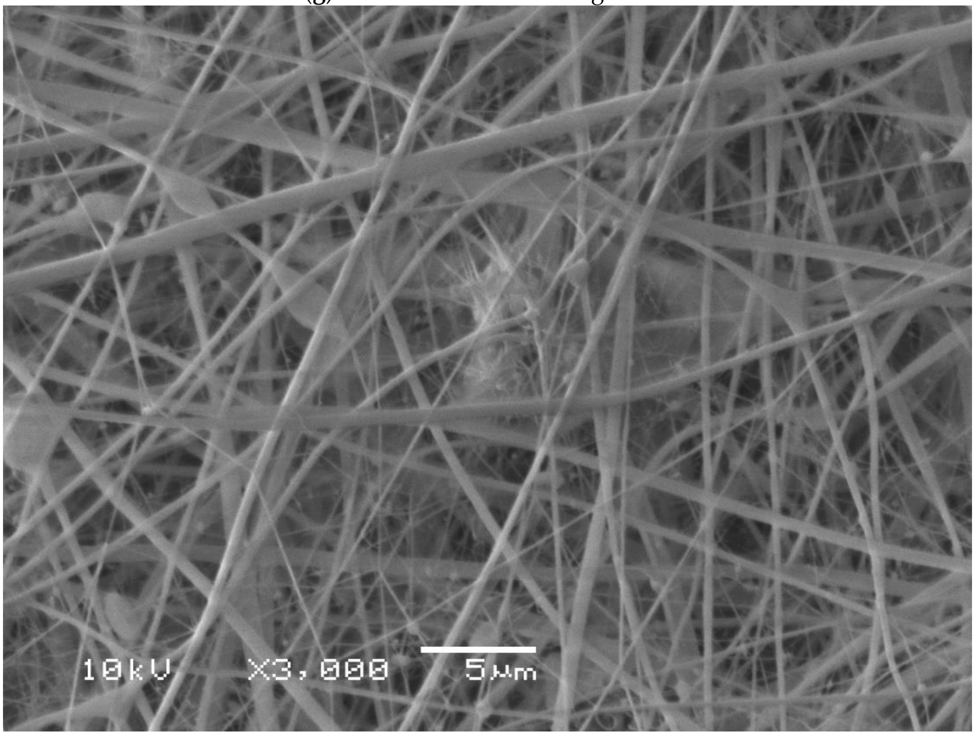

(**h**) CA13%-HA+ME 25 mg 3000x

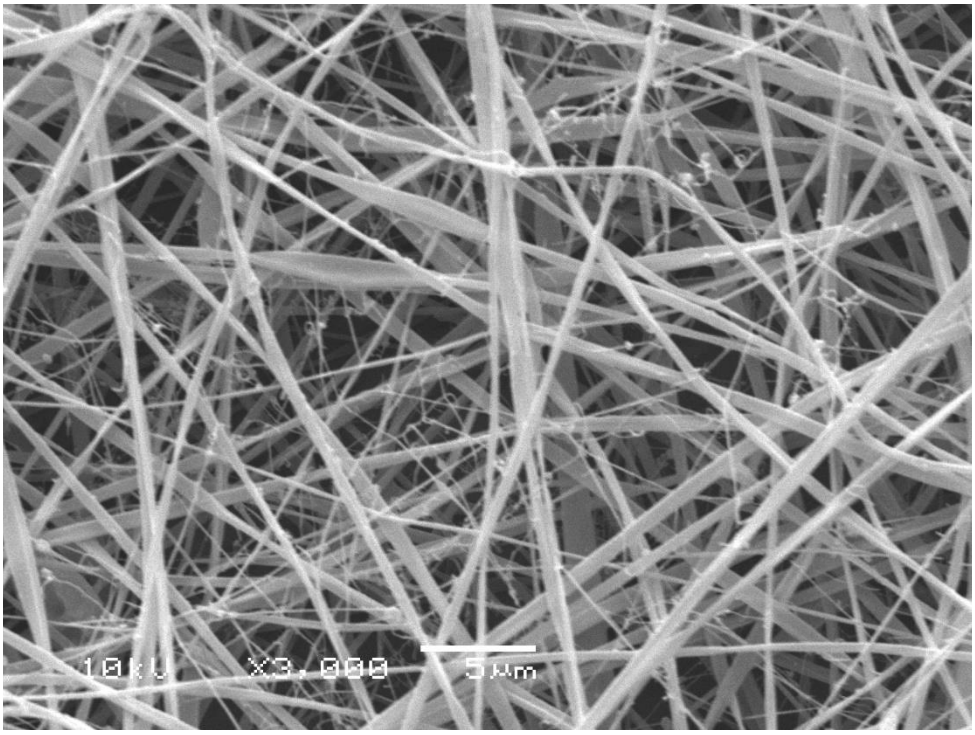

(**i**) CA13%-HA+ME 50 mg 3000x

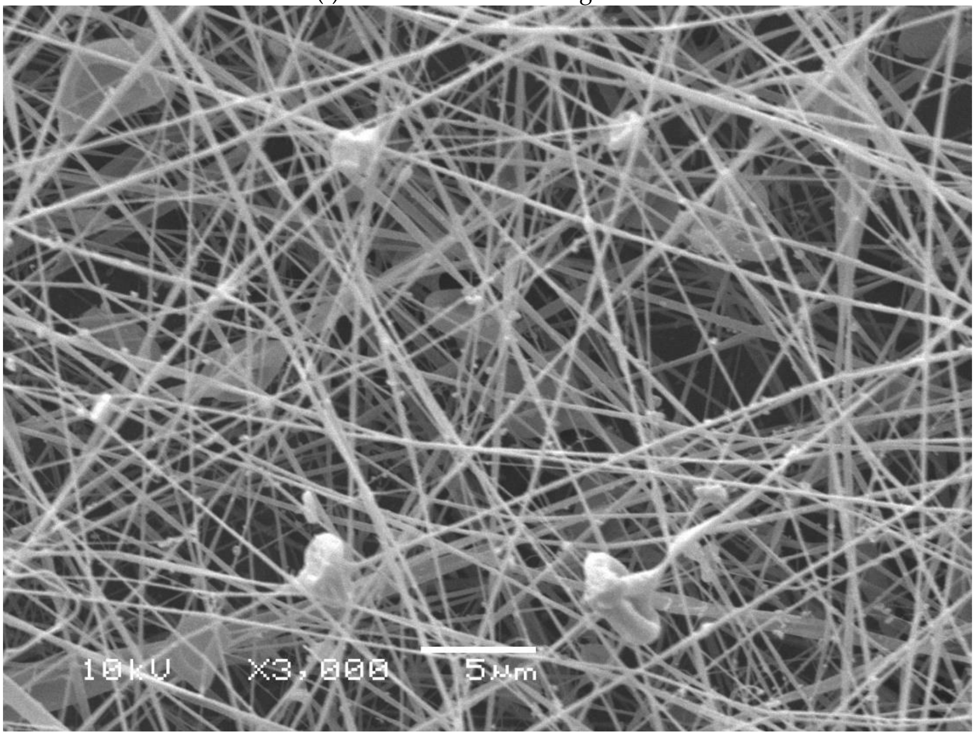

(**j**) CA13%-HA+ME 200 mg 3000x

Figure S4. SEM images of CA13/HA/ME6.25mg, CA13/HA/ME12.5mg, CA13/HA/ME25mg, CA13/HA/ME50mg, and CA13/HA/ME200mg under 1000× (**a**–**e**) and 3000× (**f**–**j**) magnifications.
